# Effectiveness and safety of acupuncture for cancer-related hiccups: a systematic review and meta-analysis

**DOI:** 10.3389/fneur.2024.1480656

**Published:** 2024-12-11

**Authors:** Zining Guo, Ying Liang, Wenhao Liu, Bingjing Huang, Huiyan Zheng, Shaoyang Cui, Nenggui Xu

**Affiliations:** ^1^Shenzhen Hospital (Fu Tian) of Guangzhou University of Chinese Medicine, Guangzhou, Guangdong, China; ^2^South China Research Center for Acupuncture and Moxibustion, Medical College of Acu-Moxi and Rehabilitation, Guangzhou University of Chinese Medicine, Shenzhen, Guangdong, China; ^3^Clinical Medical College of Acupuncture-Moxibustion and Rehabilitation, Guangzhou University of Chinese Medicine, Guangzhou, Guangdong, China

**Keywords:** acupuncture, cancer, complementary and alternative medicine, hiccups, meta

## Abstract

**Background:**

The use of acupuncture in cancer treatment is expanding. Nevertheless, the efficacy and safety of acupuncture in alleviating cancer-related hiccups remains uncertain and inconclusive.

**Methods:**

We conducted a systematic search across eight databases: PubMed, China National Knowledge Infrastructure Database, WanFang, China Science and Technology Journal Database, SinoMed, Web of Science, Cochrane, and Embase, covering the period from their inception to July 2023. Literature was screened based on predefined PICOS inclusion and exclusion criteria, and the risk of bias was assessed using the Cochrane Risk of Bias tool. Data synthesis was performed using Review Manager 5.3 software and R studio 4.4. Additionally, we conducted a frequency analysis of acupoint usage.

**Results:**

A total of nine randomized controlled trials (RCTs) involving 580 patients were included in the analysis. The risk of bias assessment indicated a high risk of bias in all nine RCTs, primarily due to blinding of participants and personnel and random sequence generation (selection bias). The meta-analysis revealed that acupuncture significantly reduced the symptoms of cancer-related hiccups compared to pharmacological treatment (relative risk (RR) = 1.83, 95% confidence interval (CI) [1.53, 2.20], *p < 0.001*, I^2^ = 27%). In terms of onset time, acupuncture demonstrated a shorter duration of onset compared to pharmacological treatment (mean difference (MD) = −8.71, 95% CI [−23.32 5.89], *p < 0.01*, I^2^ = 100%). Furthermore, acupuncture exhibited a significant improvement in sleep, eating, and appetite scores compared to pharmacological treatment (MD = 0.68, 95% CI [0.07, 1.29], *p = 0.03*; MD = 0.68, 95% CI [0.07, 1.30], *p = 0.03*; MD = 0.66, 95% CI [0.08, 1.25, *p = 0.03*]). The frequency of acupoint usage was analyzed, with ST36 and PC6 being the most frequently used acupoints. Regarding adverse events, acupuncture exhibited favorable safety profiles compared to other control groups.

**Conclusion:**

The meta-analysis results suggest that acupuncture has a positive effect on the efficacy rate for cancer-related hiccups, as well as improvements in quality of life and time to effect response. However, due to the high risk of bias and quality limitations of the included studies, no conclusive evidence currently supports the efficacy of acupuncture. High-quality, evidence-based research is still needed to confirm the effectiveness of acupuncture in treating cancer-related hiccups.

**Systematic Review Registration:**

https://www.crd.york.ac.uk/prospero/, CRD42023451403.

## 1 Introduction

Hiccups are involuntary contractions of the diaphragm and intercostal muscles resulting in sudden spasms of the glottis, a common phenomenon experienced by many individuals (1, 2). These spasms produce short, frequent sounds due to the interruption of airflow through the throat (3). While physiological hiccups resolve spontaneously, intractable hiccups persist for more than 48 h (4). Statistics indicate that 15–40% of patients with cancer experience hiccup symptoms (cancer-related hiccups) due to various reasons. Among patients with cancer receiving palliative care with significant symptom burdens, the prevalence of hiccups can be as high as 27% (5, 6). Furthermore, more than one-third of patients with these symptoms report over 1 week or longer without effective relief (7). The actual prevalence may be underestimated, as the collection of hiccup symptoms often depends on proactive inquiries by clinical managers or researchers, which may introduce recall bias. Additionally, large-scale epidemiological studies on this condition are lacking (8–10). Cancer-related hiccups not only cause uncontrollable paroxysmal spasms leading to sudden inhalation and glottis closure but may also trigger physical symptoms such as insomnia, fatigue, weakness, and malnutrition (6, 8, 11). Emotional issues like depression and irritability are also common and can easily lead to comorbidities (5, 6, 8, 11). Cancer-related hiccups are also associated with increased pain intensity, while highly proportion of patients present with cancer pain (11, 12). These issues are particularly prominent in patients with advanced cancer, who often face multiple symptom burdens and intensive treatments. Any additional illness in these patients can be fatal (approximately 9% suffering from persistent or refractory hiccups) (7, 13). The underlying cause of cancer-related hiccups is believed to be an increased excitability of the phrenic and vagus nerves (14). These hiccups may result from organic lesions, including central lesions where compression of hiccup reflex centers occurs due to intracranial and cervical tumors, and peripheral lesions where metastasis or invasion by malignant tumors affects the diaphragm or surrounding areas (15). Additionally, hiccups can be triggered by cancer treatments, electrolyte disturbances, and acid–base imbalances.

Acupuncture, a traditional Chinese medicine technique with a history of over 2000 years, involves inserting filiform needles at specific locations on the body (acupuncture points) and using twiddle and lifting techniques to treat various ailments (16). Acupuncture is widely recognized for its efficacy, convenience, affordability, and minimal side effects, making it a promising option for addressing hiccups (1). It is a significant treatment modality within traditional Chinese medicine, frequently employed in the supportive and palliative care of patients with cancer (17, 18).

The most recent systematic review/meta-analysis on acupuncture for cancer-related hiccups dates back to 2012 (19). Previous systematic reviews identified a limited number of randomized controlled trials (RCTs), many of which had a high risk of bias and insufficient supportive evidence. Therefore, it is crucial to conduct an updated systematic review and meta-analysis to assess the effect of acupuncture on cancer-related hiccups. This study aims to provide a comprehensive systematic review and meta-analysis of RCTs to evaluate the available evidence on the efficacy of acupuncture for cancer-related hiccups.

## 2 Materials and methods

This study was registered with PROSPERO (# CRD42023451403) and conducted in accordance with the Preferred Reporting Items List for Systematic Reviews (PRISMA 2020 Checklist) (20). Detailed data can be found in Supplementary Figure S2.

### 2.1 Search strategy

Two independent reviewers conducted a comprehensive search across eight databases: PubMed, The Cochrane Library, EMBASE, Web of Science, China National Knowledge Infrastructure Database, China Science and Technology Journal Database, Wan Fang Data, and China Biology Medicine, covering the period from the inception of each database until July 2023. The search strategy incorporated Medical Subject Heading terms (MeSH) and keywords. Chinese Mesh included: “Zhenci” (acupuncture), “Dianzhen” (electroacupuncture), “Xuewei” (acupoint), “Erni” (hiccup), “Liu” (tumor), “Ai” (cancer), “Hualiao” (chemotherapy), and “Fangliao” (radiotherapy). English Mesh included: “Neoplasms,” “Acupuncture,” and “Hiccups.” To ensure consistency between Chinese and English Mesh, we referenced standardized Mesh for both languages, selecting common synonyms and near-synonyms. Furthermore, two independent reviewers conducted multiple rounds of comparisons to ensure consistency. We made the necessary adjustments to the search strategy based on the requirements of individual databases. All search results were verified by a third-party reviewer, and no language restrictions were applied. The detailed search formulas for each database are presented in Supplementary Figure S1.

### 2.2 Eligibility criteria included

Studies were eligible for inclusion if they met the following criteria: (1) Involved participants with hiccups resulting from cancers (including central lesion and peripheral lesion cancers) or cancer-related treatments (including chemotherapy, radiotherapy, other medications for neoplasms, electrolyte disturbance, and acid–base imbalance treatments). (2) No limitations on age, gender, or race. (3) Involved patients with any cancer diagnosis and at any stage of disease. (4) RCTs. (5) The intervention group received acupuncture as a sole treatment. Acupuncture was defined as piercing the skin with a needle. (6) The control group was either a blank, placebo, or group receiving other conventional treatments or medical treatment. (7) The primary outcome measure was the overall effectiveness rate, and the secondary outcome was the rate of adverse events, on set time, quality of life (QoL).

### 2.3 Exclusion criteria included

Studies were excluded if they met the following: (1) non-human studies or animal cell experiments. (2) Studies with intervention measures not meeting the inclusion criteria. (3) Literature reviews, abstracts, personal academic opinions, conference reports, and case reports. (4) Studies with incomplete outcome indicators or outcome indicators that could not be extracted. (5) Duplicate publications or studies with overlapping data. (6) Studies where full text was unavailable. All excluded studies presented in Supplementary File S1.

### 2.4 Data extraction

All articles were read by two independent reviewers (HYZ and BJH), who extracted data from the articles according to predefined criteria. If a dispute arose, a third researcher (RL) decided whether to include or exclude the article. The extracted data included article titles, author names, publication years, participant characteristics, hiccup types, concomitant diseases, intervention details, and comparisons. Two researchers (HYZ and BJH) cross-checked the data extraction. In case of disagreements, the third researcher (YL) was involved. We contacted the authors for further information if necessary.

### 2.5 Risk of bias

Two independent reviewers (ZNG and HYZ) used the Cochrane risk-of-bias tool (ROB 1.0) independently to assess the methodological quality of the included RCTs, with discrepancies addressed through discussion with a third reviewers (21).

### 2.6 Synthesis of data

Data were synthesized using R Studio 4.4 with the “meta” package and Review Manager 5.3 software. For continuous outcomes, the effect size was measured as the mean difference ± standard deviation (MD ± SD), while for dichotomous outcomes, the effect size was calculated as relative risk (RR). The corresponding 95% confidence intervals (CIs) were calculated. Heterogeneity between trials was assessed using the chi-square (χ^2^) test, and the I^2^ statistic was used to quantify the degree of heterogeneity. A random-effects model was applied for pooling when I^2^ > 50% and *p > 0.1*, indicating significant heterogeneity; otherwise, a fixed-effects model was applied (21). Depending on the degree of heterogeneity, we aim to identify potential sources of heterogeneity through pre-planned subgroup analyses (according to acupuncture type) or meta-regression. In addition, we will also perform a sensitivity analysis by excluding each included study one by one in the meta-analysis based on the results of the meta-analysis and the final choice of model (fixed effect/random effect model) to determine changes in significance, effect size, and heterogeneity to clarify the reliability of the results.

## 3 Results

**Figure 1:**
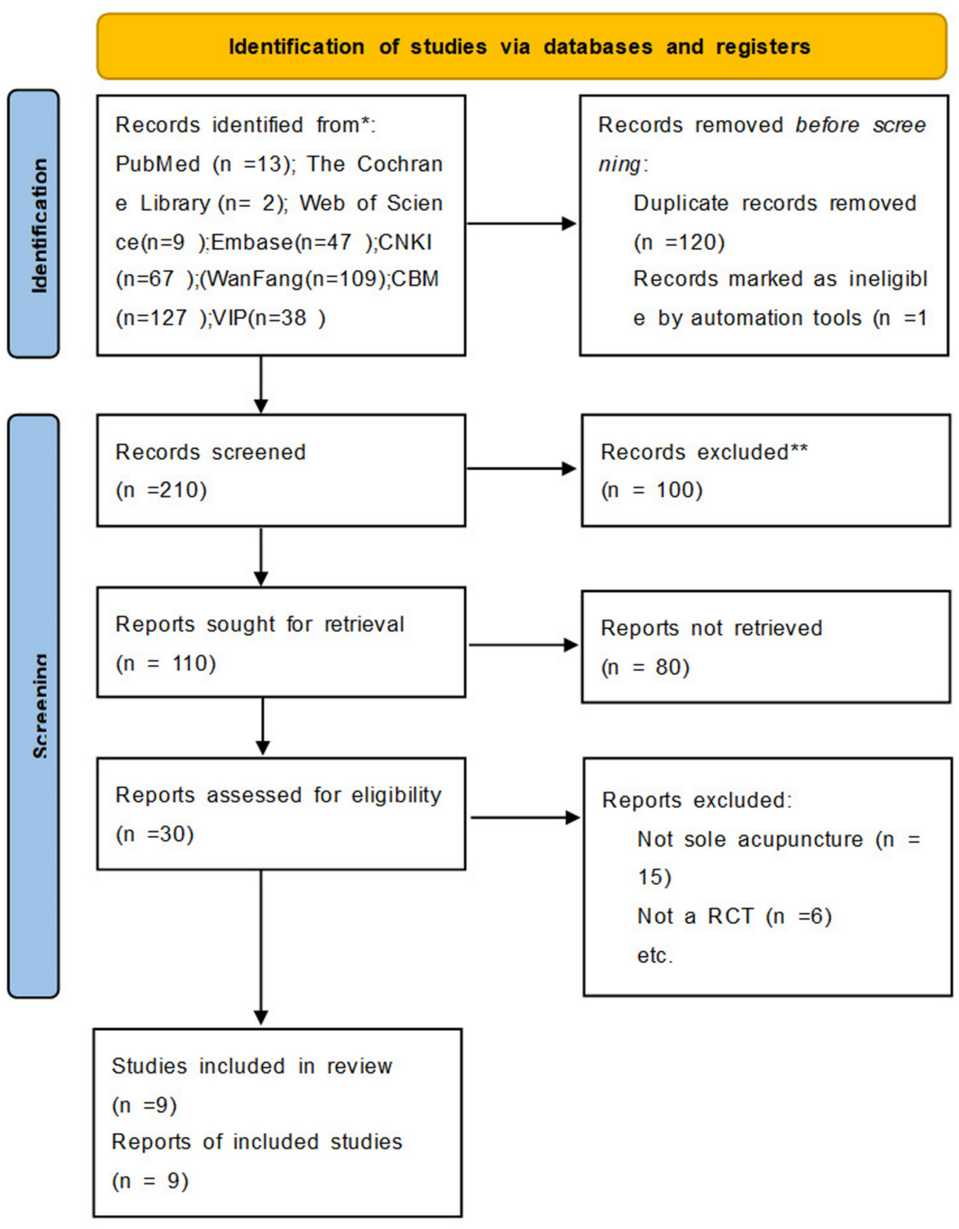
Flowchart of study selection.

Initially, 355 articles were identified through our search strategy. After reviewing the titles and abstracts, 100 articles were excluded, leaving 30 articles for full-text screening. Ultimately, nine studies encompassing a total of 580 participants were deemed suitable for qualitative and quantitative analysis (Figure 1) (22–30).

### 3.1 Study characteristics

A total of 9 studies, involving 580 patients, were included in the analysis. Among these, 293 patients received acupuncture or related treatments, while 287 patients were assigned to control groups Table 1 (22–30). The mean age of the included patients ranged from 25 to 78, and all participants were diagnosed with cancer. The studies employed four different acupuncture techniques, namely manual acupuncture (MA), electroacupuncture (EA), ear acupuncture, and pressing needling. The control treatments in the control groups consisted of oral administration of Baclofen and metoclopramide muscle injection. Among the included studies, the most frequently utilized acupoints were Zusanli (ST 36) and Neiguan (PC 6) which were featured in seven of the studies. ST 36 belongs to the foot Yangming stomach channel, while PC 6 belongs to the foot Taiyin spleen channel. Four studies compared the efficacy of MA alone with antispasmodic drug therapy, while three studies compared EA monotherapy with drug injections. One study compared pressing needling with metoclopramide muscle injection, and another study compared ear acupuncture with oral Baclofen and breath-holding.

**Table 1:** Characteristics of the included studies.

Author	Year	Country	Age	Randomization	Cancer type	Intervention	Control	Mainly acupuncture points	Acupuncture session	Time	Primary outcomes
Yinghua Li	2016	China	49.8 ± 7.8	Not specifically reported	Liver Cancer	MA	Baclofen Tablets	PC6, LR3, DU26, RN22, LI4, BL2	7 sessions	30 min	ER
Guohua Fan	2021	China	58.43 ± 6.86	Random number table	Lung Cancer	QA	Conventional care	HX1, TF4, CO15	7 sessions	24 h	ER, KPS
Chenchen Wang	2022	China	52.23 ± 1.43	Not specifically reported	Stomach Cancer	EA	Antiemetic	ST36, BL17, PC6	14 sessions	20 min	ER, Onset Time, KPS
Shike Zhang	2018	China	48.02 ± 5.31	Random number table	Liver Cancer	EA	Antiemetic	CO11, CO12, CO13, CO4, CO17	6 sessions	30 min	ER, KPS
Yong Zhang	2017	China	Not specifically reported	Random number table	Liver Cancer	AA	Baclofen Tablets	PC6, LR3, DU26, RN22, LI4, BL2	5 sessions	30 min	ER
Weixiang Xue	2011	China	Not specifically reported	Order of Consultation	Liver Cancer	MA	Antiemetic	LR3, SP04, CV17, ST36, PC6	10 sessions	30 min	ER
Xuemei Liu	2007	China	Not specifically reported	Random number table	Liver Cancer	MA	Sedative drug	RN22, ST36, PC6	3 sessions	30 min	ER
Hongtao Chen	2006	China	51.37 ± 11.10	Order of Consultation	Liver Cancer	MA	Antiemetic	Bilateral PC6, ST36, CV17, BL2	10 sessions	30 min	ER
Xianjun Liu	2015	China	52.4 ± 10.5	Order of Consultation	Lung Cancer	MA	Baclofen Tablets	LR03, LI4, BL2, PC6, RN22	7 sessions	30 min	ER

### 3.2 Risk of bias in individual trials

**Figure 2:**
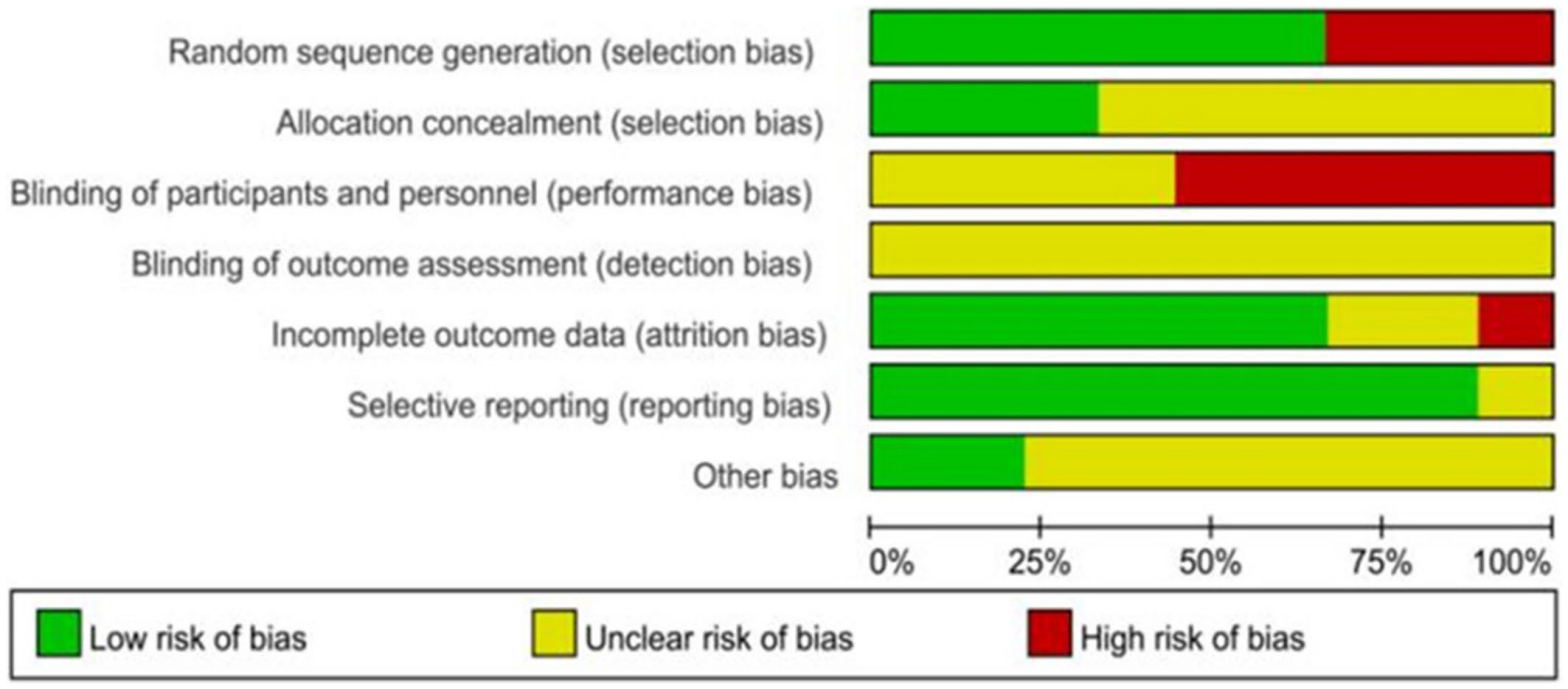
Individual risk of the bias.

**Figure 3:**
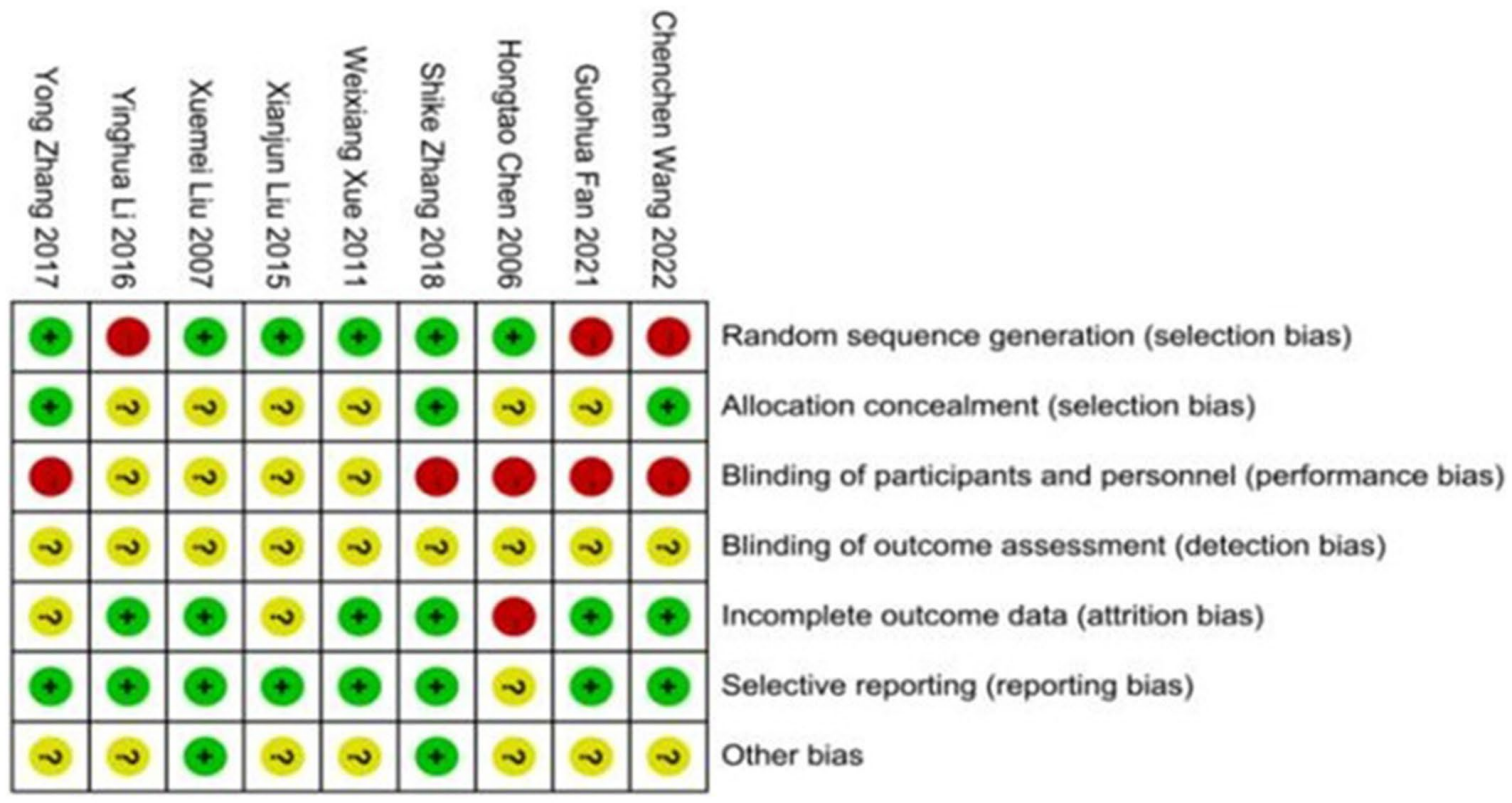
Risk of bias summary.

The overall risk of bias is presented in Figures 2, 3. The major sources of risk of bias were allocation concealment and blinding of participants and personnel (performance bias). The individual risk of bias for each trial is presented in (Figure 3). Five trials had a high risk of bias (primarily due to the blinding of participants and personnel). In comparison, nine trials had a relatively low risk of bias (mainly due to the random sequence generation). None of the studies reported the entity that measured the outcome, so the risk of detection bias was considered unclear. Additionally, none of the trials reported sample size calculation (22–29, 31).

### 3.3 Analysis of effective rate

**Figure 4:**
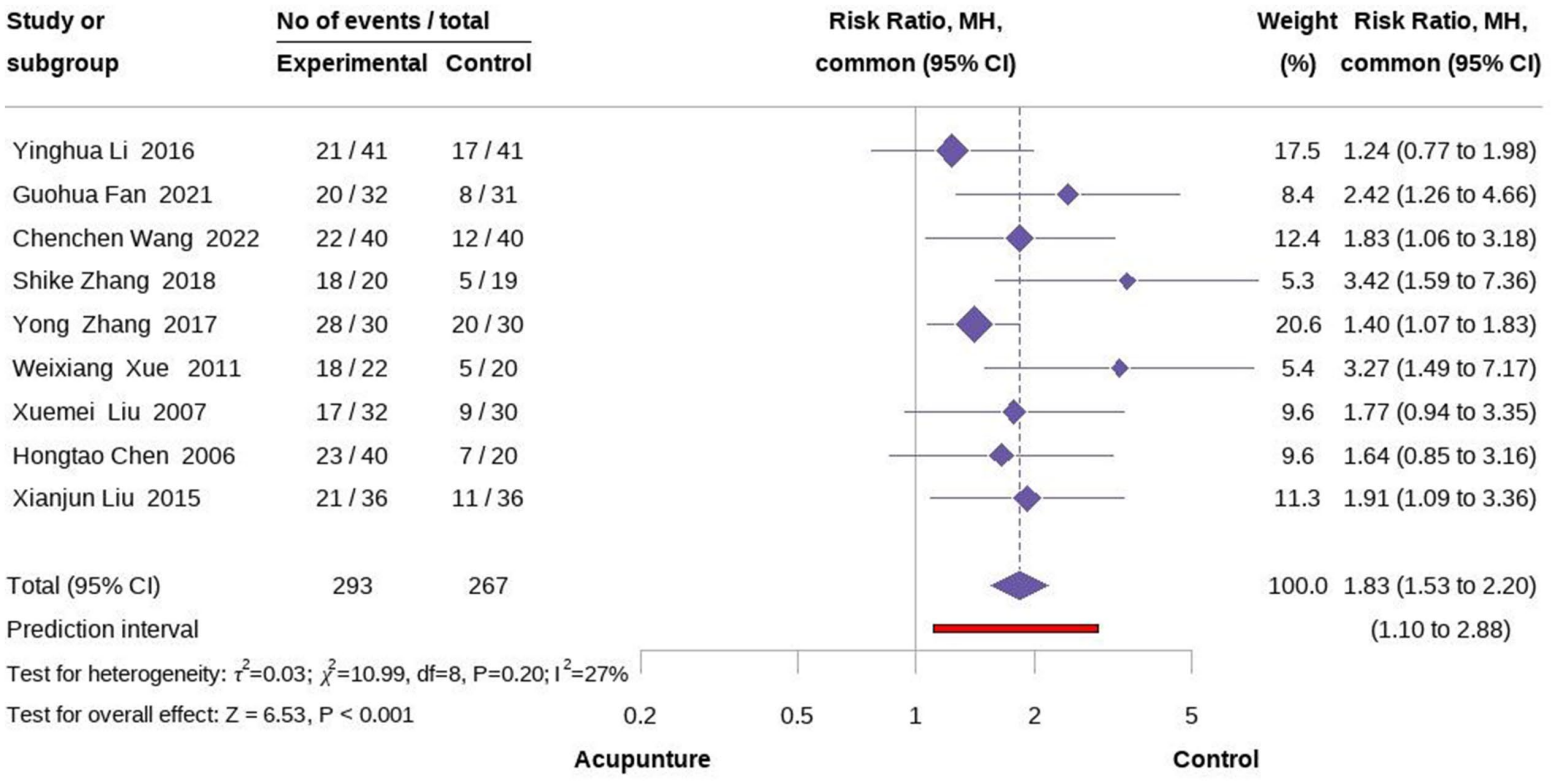
Forest plot of overall effectiveness rate.

**Figure 5:**
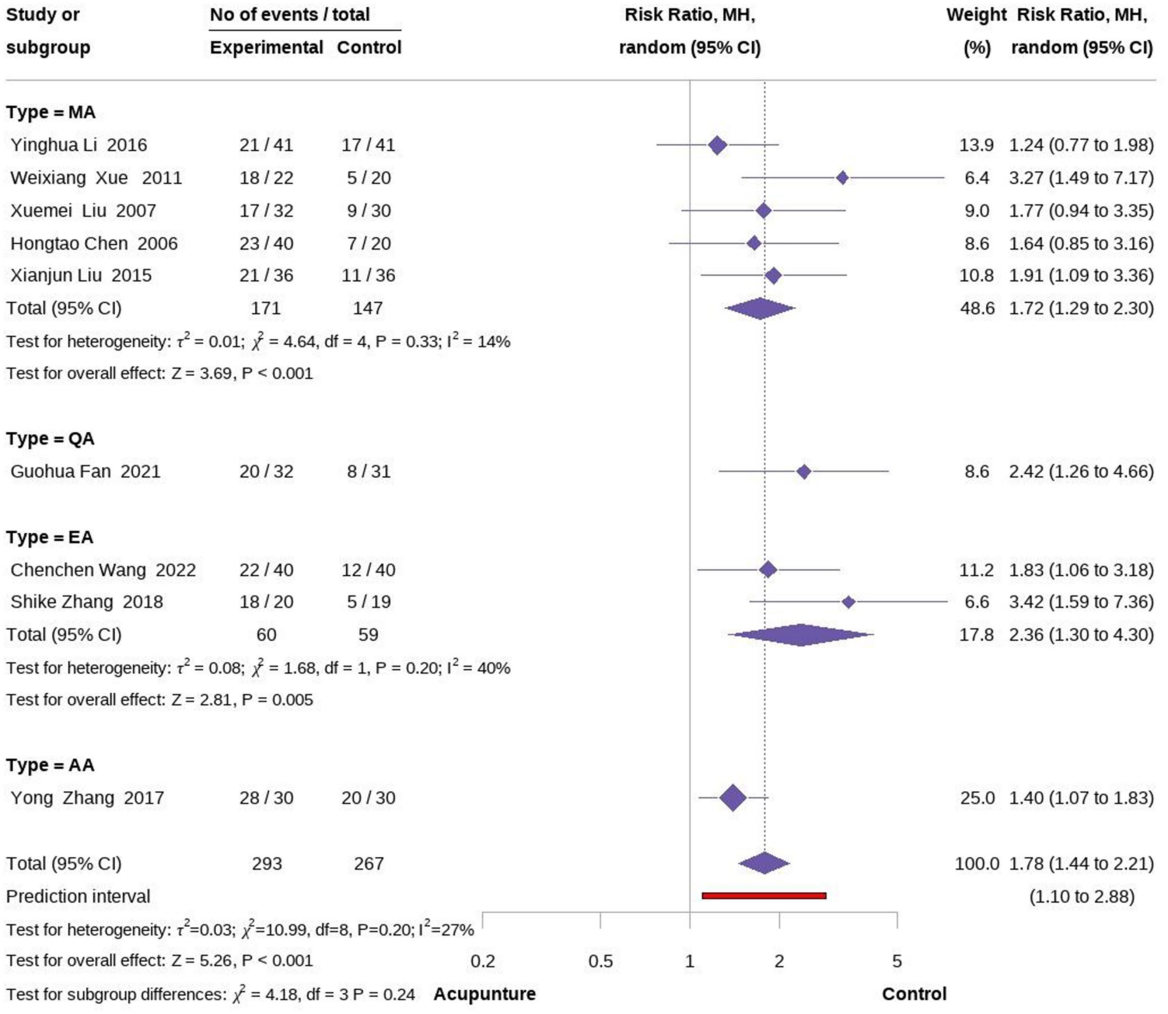
Forest plot of subgroup according to acupuncture type.

In terms of effective rate, nine studies involving 560 participants compared the effectiveness of acupuncture with medication in relieving cancer-related hiccup symptoms (22–30). The meta-analysis of the nine studies (Fixed model) demonstrated that acupuncture had a superior efficacy rate in treating cancer-related hiccups compared to oral and intramuscular medication, with a statistical difference (RR = 1.83, 95% CI [1.53, 2.20], *p < 0.001*, I^2^ = 27%). To further explore whether different acupuncture techniques yielded varying effects on the treatment of cancer-related hiccups, we conducted a subgroup analysis based on the type of acupuncture. The results indicated that EA was more effective than MA, although the EA group also exhibited higher heterogeneity than MA (RR =2.36, 95% CI [1.30, 4.30], *p* = 0.005, I^2^ = 40%; RR =1.72, 95% CI [1.29, 2.30], *p < 0.001*, I^2^ = 14%;) (Figures 4, 5).

### 3.4 Analysis of other outcome measures

**Figure 6:**
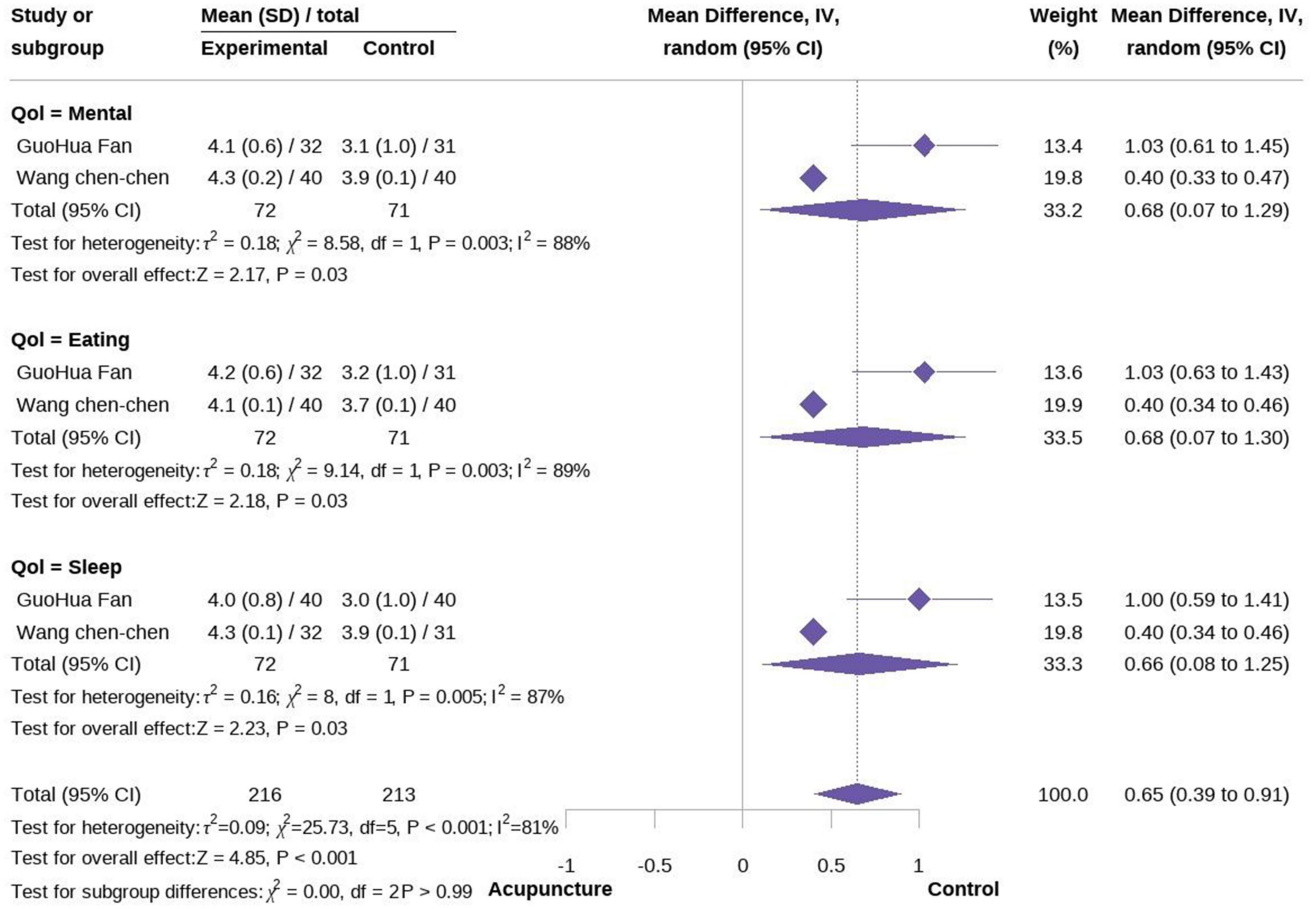
Forest plot of quality of life (mental, eating, and sleep).

**Figure 7:**
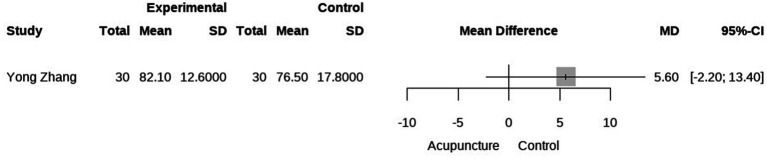
Forest plot of KPS.

**Figure 8:**
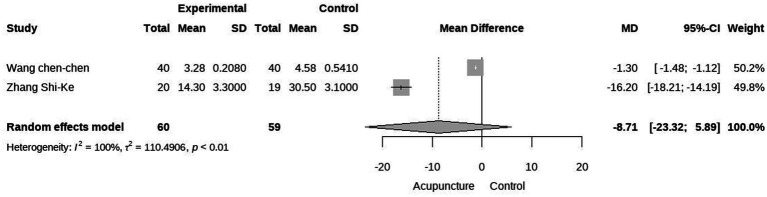
Forest plot of onset time.

Secondary outcomes included QoL and time to treatment response. Three studies evaluated the impact of acupuncture on QoL in cancer-related hiccups (23, 24, 27). Two of these studies used QoL assessment tools focusing on mental well-being, eating, and sleep, while one used the Karnofsky Performance Status (KPS) score. A meta-analysis of mental, diet, and sleep QoL demonstrated that acupuncture was more effective than pharmacological treatment in improving these aspects, although there is high heterogeneity (MD = 0.68 95% CI [0.07, 1.29], *p* = 0.03, I^2^ = 88%; MD = 0.68 95% CI [0.07, 1.30], *p = 0.03*, I^2^ = 89%; MD = 0.66 95% CI [0.08, 1.25], *p = 0.03*, I^2^ = 87%; Figures 5, 6). We conducted a meta-regression to identify potential sources of heterogeneity, and the results showed that the type of acupuncture was a potential source of heterogeneity (*p* < 0.05, see Appendix). Regarding KPS, acupuncture was found to improve KPS scores compared to pharmacological treatment alone (MD = 5.60, 95% CI [13.40], Figure 7). Additionally, a meta-analysis of treatment response time indicated that acupuncture led to a faster onset of effect compared to pharmacological treatment. However, due to the limited number of studies, we were unable to further identify the sources of heterogeneity, which we speculate may be attributed to differences in study design (MD = −8.71, 95% CI [−23.32; 5.89], *p* < 0.01, I^2^ = 100%) (Figure 8).

### 3.5 Adverse events

Five studies reported acupuncture-related adverse reactions. Four studies indicated that there were no adverse reactions in the acupuncture group after treatment (22–24, 27, 28). One study reported that a patient in the acupuncture group experienced mild pain. Overall, acupuncture was found to be relatively safe, with no serious adverse events (SAEs).

### 3.6 Sensitivity analysis

We conducted a sensitivity analysis for the meta-analysis of effectiveness rate, QoL and onset time by sequentially excluding studies according to a predefined strategy. After examining the effect size, significance, and heterogeneity, the results were confirmed to be robust (Supplementary File S2).

## 4 Discussion

### 4.1 Summary the key finding

Overall, the meta-analysis of acupuncture for cancer-related hiccups demonstrated positive effects compared to pharmacological treatments, specifically in terms of efficacy, QoL, and time to treatment response. A particularly notable impact was observed in efficacy, with a moderate effect size (RR = 1.83, 95% CI [1.53, 2.20], *p* < *0.001*, I^2^ = 27%) (22–30). Additionally, the safety of acupuncture, which has been validated in previous studies, was confirmed in this context. Among 158 patients, only one reported mild pain, and no SAEs were observed (22–30). However, it is important to note that all nine studies included in this analysis exhibited a high risk of bias, and the overall quality of the studies was suboptimal. Consequently, the results of this meta-analysis should be interpreted with caution. Further high-quality, evidence-based studies are needed to clarify the effectiveness of acupuncture in treating cancer-related hiccups.

The impact risk of bias is particularly prominent in our meta-analysis. Using the Risk of Bias tool, we identified allocation concealment and blinding as the main sources of bias. Nearly all of the included studies failed to report whether allocation concealment, such as opaque envelopes, was used. This reflects the need for greater rigor in acupuncture clinical research (32). In RCTs, proper allocation concealment is crucial. If allocation concealment is inadequate, there is a risk that those responsible for randomization may have prior knowledge of group assignments, leading to “subjective selection,” where individuals more suitable for acupuncture intervention are preferentially assigned to the intervention group, potentially leading to an overestimation of the effects of acupuncture (21, 33, 34). Furthermore, if participants are aware of their group assignment before the intervention, those in the control group may have reduced expectations. This could lead to an underestimation of the effects of acupuncture during outcome measurement. This issue is particularly evident in the small-sample RCTs included in our meta-analysis, raising concerns about the reliability of the results. Regarding blinding, due to the unique nature of acupuncture, it is difficult to blind acupuncturists (32, 35). Therefore, this limitation must be addressed through other aspects of study design. Unfortunately, the importance of this issue was not adequately acknowledged in the included studies, and nearly all failed to report whether outcome assessors were blinded or provided information regarding statisticians. This oversight can lead to consequences similar to those resulting from poor allocation concealment, overestimation, or underestimation of acupuncture effects, resulting in unreliable outcomes. As emphasized earlier, due to these bias risks, the interpretation of our results should be approached with caution. Furthermore, heterogeneity remains a significant challenge in this study. Although subgroup analysis and meta-regression were conducted to identify some sources of heterogeneity, substantial heterogeneity persists. We speculate that this may be due to factors such as differences in tumor types, acupuncture intervention frequencies, and other aspects of study design.

### 4.2 Implication for practice

In recent years, the application of acupuncture therapy has steadily expanded across a variety of clinical areas (34, 36). The World Health Organization reports that acupuncture is now practiced in over 103 countries globally (34). In oncology, acupuncture has been incorporated into clinical practice guidelines for managing symptoms such as cancer pain and hot flashes in breast cancer, highlighting its potential and promising prospects for treating cancer-related symptoms (37, 38). For cancer-related hiccups, pharmacological treatments, including Baclofen, dexamethasone, and gabapentin, remain the standard of care (39, 40). However, these medications often lead to adverse reactions, such as dizziness and excessive sedation, which can complicate treatment for patients with cancer who have already undergone multiple therapies (39–41). This highlights the urgent need to explore effective, safe, and cost-efficient non-pharmacological interventions as part of personalized treatment approaches (42, 43). In our meta-analysis, we found evidence supporting the use of non-pharmacological interventions, particularly acupuncture, for treating cancer-related hiccups. Compared to pharmacological treatment alone, acupuncture demonstrated positive effects, including improved efficacy and QoL. Notably, the calculated minimal clinically important difference using the distribution method (0.5 standard deviations), acupuncture also exceeded the threshold (44). However, the limitations of the included studies, such as their low quality, should not be overlooked. Therefore, future research can focus on well-designed, evidence-based acupuncture RCTs, following the pathway of exploratory research, explanatory research, and confirmatory research, to establish the efficacy of acupuncture in treating cancer-related hiccups. Additionally, there is a lack of visualizable and interpretable evidence regarding the mechanisms by which acupuncture treats cancer-related hiccups. Preliminary suggest that acupuncture may improve blood flow to the hypothalamus, with bidirectional regulation of gastrointestinal motility, potentially helping to alleviate hiccups (45). Acupuncture also influences the neuroendocrine system by stimulating nerve endings at acupuncture points, thereby promoting the production and secretion of neurotransmitters such as norepinephrine, serotonin, and *γ*-aminobutyric acid, which can inhibit the occurrence of hiccups (46). Additionally, evidence suggests that acupuncture can activate the cerebral cortex, suppress abnormal excitation of the vagus nerve, and ultimately relieve diaphragmatic spasms (47). Some studies also indicate that specific acupuncture points such as ST 36 and PC 6 may stimulate neurons in the solitary tract nucleus of rats with gastrointestinal motility disorders (48). This mechanism is believed to improve gastrointestinal motility through the somatic-visceral pathway. It is worth noting that among the nine studies included in this research, five employed the PC6 point (23, 25, 26, 29, 30). In traditional Chinese medicine, the PC6 point is commonly used to regulate the spleen and stomach qi and to settle reversed stomach qi, making it a popular point for treating hiccups (49). However, the underlying neurobiological mechanisms of acupuncture at the PC6 point for treating cancer-related hiccups remain unclear, and further research is needed to explore and validate these mechanisms. Additionally, a key area for improvement in the acupuncture clinical system is the development of an evidence-based risk–benefit system. Currently, data on the risks of acupuncture primarily stems from adverse events (AEs). Common AEs can be classified into four main categories: (1) tissue damage, such as bleeding, (2) infection from needles or other causes, such as inflammation, (3) local adverse reactions, such as pain, and (4) other reactions, such as dizziness, among others (50). While most studies acknowledge that minor AEs, such as bleeding and pain, account for a large proportion and generally resolve spontaneously without treatment (51), recent data suggests that there are only 1–7 minor AEs per 1,000 patients (50); however, risk factors, such as improper handling by acupuncturists, may lead to SAEs. The most classic of these is traumatic SAE from needling, resulting in symptoms such as pneumothorax, pericardial effusion, neuropathy, and even visceral hemorrhage (51, 52). The occurrence and management of SAEs present significant challenges for clinical decision-makers, patients, and the public. This is particularly true for patients with cancer, who often carry a high symptom burden, for whom any SAEs can be fatal. Moreover, there is no standardized system for determining acupuncture-related AEs, and most RCTs rely on the World Health Organization-Uppsala Monitoring Center criteria or other tools to assess the causal relationship between acupuncture and AEs (53). Given the specificity of acupuncture, this approach may lead to an inevitable mis-assessment of the number of acupuncture-related AEs, which lacks a certain persuasive power of evidence in the context of evidence-based medicine, particularly when integrating acupuncture clinical trials. We believe that addressing the risks of AEs alongside rigorous RCTs demonstrating efficacy and ultimately constructing an evidence-based risk–benefit system will help enhance the influence of acupuncture (54, 55). Additionally, our study found that there is currently no unified diagnostic standard or clinical practice guideline for cancer-related hiccups. We urge the establishment of relevant standards and treatment guidelines in future research to provide more reference information for clinical treatment and studies.

### 4.3 Limitations and advantages

Our meta-analysis has several limitations. First, the quality of the included studies was relatively low, with a high risk of bias and small sample sizes, which could lead to either overestimation or underestimation of acupuncture effects. Second, despite performing heterogeneity analysis, significant heterogeneity remains among the included studies. Lastly, sham acupuncture was not sufficiently utilized in the included studies, and acupuncture continues to be challenged by the placebo effect. Eliminating non-specific effects remains a major challenge. Compared to previous meta-analyses, our study offers some advantages. The last meta-analysis on this topic was conducted in 2012, making an update necessary. In contrast, we successfully included four new studies and performed the analysis using standard methods. Additionally, we followed the PRISMA guidelines for reporting. Furthermore, we have provided a more in-depth analysis of the existing issues, aiming to provide further insights for future research on acupuncture for cancer-related hiccups.

## 5 Conclusion

The results of this meta-analysis suggest that acupuncture has a positive effect on the efficacy rate for cancer-related hiccups, as well as improvements in QoL and time to effect response. However, due to the high risk of bias and quality limitations of the included studies, conclusive evidence establishing the efficacy of acupuncture is not yet available. High-quality, evidence-based research is still required to confirm the effectiveness of acupuncture in treating cancer-related hiccups.

## Data Availability

The original contributions presented in the study are included in the article/Supplementary material, further inquiries can be directed to the corresponding authors.
