# Supplementary material for: Effectiveness and safety of acupuncture for cancer-related hiccups: a systematic review and meta-analysis
Source: Front Neurol. 2024 Dec 11;15:1480656. doi: 10.3389/fneur.2024.1480656 (PMC11668674; doi:10.3389/fneur.2024.1480656)
Supplement: Supplementary file 1 [file Supplementary_file_1.docx]

# Search strategy

# 1 CNKI

Search strategy

(摘要=RCT + 随机对照 + 随机) AND (主题=肿瘤 + tumor spread + tumore + tumors + tumour + 瘤子 + 瘤 + 癌) AND (主题=针刺 +针灸 + 针法 + 体针 + 电针 + 穴位）AND（主题=呃逆 + hiccup + 打嗝 + 哕）

# 2 VIP

Search strategy

摘要:RCT+随机对照+随机 AND 题名或关键词:呃逆+hiccup+打嗝+哕 AND 题名或关键词:肿瘤+tumor spread+tumore+tumors+tumour+瘤子+瘤+癌 AND 题名或关键词:针刺+针灸+针法+体针+电针+穴位

# 3 CBM

Search strategy

("呃逆"[标题:智能] OR "打嗝"[摘要:智能] OR "哕"[摘要:智能] OR "hiccup"[摘要:智能]) AND ("肿瘤"[标题:智能] OR "癌症"[摘要:智能] OR "瘤"[摘要:智能] OR "癌症晚期"[摘要:智能]) AND ("针灸"[标题:智能] OR "针刺"[摘要:智能] OR "体针"[摘要:智能] OR "毫针"[摘要:智能])

# 4 WanFang

Search strategy

((主题=(呃逆 OR hiccup OR 打嗝 OR 哕)) AND 主题=(肿瘤 OR tumor spread OR tumore OR tumors OR tumour OR 瘤子 OR 瘤 OR 癌)) AND 摘要=(RCT OR 随机对照 OR 随机)) AND 主题=(针刺 OR 针灸 OR 针法 OR 体针 OR 电针 OR 穴位)

# 4 Web of Science

Search strategy

((TS=(acupuncture)) AND TS=(hiccup)) AND TS=(neoplasm)

# 5 Embase

Search strategy

Session Results

.......................................................

No. Query Results Results Date

#10. #7 AND #8 AND #9 30 5 Jul 2023

#9. #5 OR #6 67,812 5 Jul 2023

#8. #3 OR #4 6,886,772 5 Jul 2023

#7. #1 OR #2 5,110 5 Jul 2023

#6. 'acupressure':ab,ti OR 'acupuncture 11,926 5 Jul 2023

analgesia':ab,ti OR 'auricular acupuncture':ab,ti

OR 'catgut embedding':ab,ti OR

'electroacupuncture':ab,ti OR

'pharmacopuncture':ab,ti OR 'warm

acupuncture':ab,ti

#5. 'acupuncture'/exp OR 'acupuncture' 67,619 5 Jul 2023

#4. 'tumor':ab,ti OR 'tumors':ab,ti OR 4,659,529 5 Jul 2023

'neoplasm':ab,ti OR 'neoplasia':ab,ti OR

'neoplasias':ab,ti OR 'cancer':ab,ti OR

'cancers':ab,ti OR 'malignant neoplasm':ab,ti OR

'malignancy':ab,ti OR 'malignancies':ab,ti OR

'malignant neoplasms':ab,ti OR 'neoplasm,

malignant':ab,ti OR 'neoplasms, malignant':ab,ti

OR 'benign neoplasms':ab,ti OR 'benign

neoplasm':ab,ti OR 'neoplasms, benign':ab,ti OR

'neoplasm, benign':ab,ti OR 'chemotharapy':ab,ti OR 'Radiotherapy':ab,ti

#3. 'neoplasm'/exp OR 'neoplasm' 6,025,883 5 Jul 2023

#2. 'hiccups':ab,ti OR 'hiccough':ab,ti OR 2,087 5 Jul 2023

'hiccoughs':ab,ti

#1. 'hiccup'/exp OR 'hiccup' 4,804 5 Jul 2023

# 6 PubMed

Search strategy

((((Acupuncture[MeSH Terms])) OR ((Pharmacopuncture[Title/Abstract]) OR (Acupuncture Treatment[Title/Abstract]) OR (Acupuncture Treatments[Title/Abstract]) OR (Treatment, Acupuncture[Title/Abstract]) OR (Therapy, Acupuncture[Title/Abstract]) OR (Pharmacoacupuncture Treatment[Title/Abstract]) OR (Treatment, Pharmacoacupuncture[Title/Abstract]) OR (Therapy, Pharmacoacupuncture[Title/Abstract]) OR (Pharmacoacupuncture Therapy[Title/Abstract]) OR (Acupotomy[Title/Abstract]) OR (Acupotomies[Title/Abstract]))) AND (("Neoplasms"[Mesh]) OR ((Tumor[Title/Abstract]) OR (Neoplasm[Title/Abstract]) OR (Tumors[Title/Abstract]) OR (Neoplasia[Title/Abstract]) OR (Neoplasias[Title/Abstract]) OR (Cancer[Title/Abstract]) OR (Cancers[Title/Abstract]) OR (Malignant Neoplasm[Title/Abstract]) OR (Malignancy[Title/Abstract]) OR (Malignancies[Title/Abstract]) OR (Malignant Neoplasms[Title/Abstract]) OR (Neoplasm, Malignant[Title/Abstract]) OR (Neoplasms, Malignant[Title/Abstract]) OR (Benign Neoplasms[Title/Abstract]) OR (Benign Neoplasm[Title/Abstract]) OR (Neoplasms, Benign[Title/Abstract]) OR (Neoplasm, Benign[Title/Abstract])))) AND ((Hiccups[Title/Astract]) OR (Hiccough[Title/Astract]) OR (Hiccoughs[Title/Astract]) )

# 7 Cochrane Library

Search strategy

ID Search Hits

#1 (Hiccups):ti,ab,kw OR (Hiccough):ti,ab,kw OR (Hiccoughs):ti,ab,kw 155

#2 (Neoplasms):ti,ab,kw OR (Malignant):ti,ab,kw OR (Cancers):ti,ab,kw OR (MalignantNeoplasm):ti,ab,kw OR (Neoplasm):ti,ab,kw OR (Malignant):ti,ab,kw OR (MalignantNeoplasms):ti,ab,kw OR (Malignancies):ti,ab,kw OR (Malignancy):ti,ab,kw OR (Cancer):ti,ab,kw OR (BenignNeoplasms):ti,ab,kw OR (BenignNeoplasm):ti,ab,kw OR (Benign):ti,ab,kw OR (Neoplasms):ti,ab,kw OR (Neoplasm):ti,ab,kw OR (Benign):ti,ab,kw OR (Neoplasias):ti,ab,kw OR (Tumors):ti,ab,kw OR (Neoplasia):ti,ab,kw OR (Tumor):ti,ab,kw OR (Neoplasm):ti,ab,kw OR (Chemotharapy):ti,ab,kw OR (Radiotherapy):ti,ab,kw 261618

#3 MeSH descriptor: [Acupuncture] explode all trees 713

#4 MeSH descriptor: [Neoplasms] explode all trees 111738

#5 (Tumor):ti,ab,kw OR (Neoplasm):ti,ab,kw OR (Tumors):ti,ab,kw OR (Neoplasia):ti,ab,kw OR (Neoplasias):ti,ab,kw OR (Cancer):ti,ab,kw OR (Cancers):ti,ab,kw OR (Malignant Neoplasm):ti,ab,kw OR (Malignancy):ti,ab,kw OR (Malignancies):ti,ab,kw OR (Malignant Neoplasms):ti,ab,kw OR (Neoplasm, Malignant):ti,ab,kw OR (Neoplasms, Malignant):ti,ab,kw OR (Benign Neoplasms):ti,ab,kw OR (Benign Neoplasm):ti,ab,kw OR (Neoplasms, Benign):ti,ab,kw OR (Neoplasm, Benign):ti,ab,kw 241584

#6 (Pharmacopuncture):ti,ab,kw OR (Acupuncture Treatment):ti,ab,kw OR (Acupuncture Treatments):ti,ab,kw OR (Treatment, Acupuncture):ti,ab,kw OR (Therapy, Acupuncture):ti,ab,kw OR (Pharmacoacupuncture Treatment):ti,ab,kw OR (Treatment, Pharmacoacupuncture):ti,ab,kw OR (Pharmacoacupuncture Therapy):ti,ab,kw OR (Therapy, Pharmacoacupuncture):ti,ab,kw OR (Acupotomy):ti,ab,kw OR (Acupotomies):ti,ab,kw 15110

#7 MeSH descriptor: [Neoplasms] explode all trees 111738

#8 #2 OR #4 274331

#9 #3 OR #6 15177

#10 #8 and #9 1507

#11 MeSH descriptor: [Hiccup] explode all trees 55

#12 #1 OR #11 192

#13 #12 AND #9 AND #8 2

Included and excluded studies

Below are the 355 papers we included, including nine inclusion papers and the reasons for exclusion of 346 papers

| Number | **CBM Title** | Exclude Reason |  |
| --- | --- | --- | --- |
| 1 | Progress of clinical research on the improvement of gastrointestinal adverse effects of chemotherapy by acupuncture and moxibustion | Review |  |
| 2 | Effect of spleen-strengthening and stomach-healing soup combined with warm acupuncture in the treatment of chronic atrophic gastritis of spleen deficiency and cold-fearing type and its effect on the level of inflammatory factors | Subjects were not tumour patients |  |
| 3 | Clinical study on the treatment of diabetic gastroparesis with “standardized matching points” acupuncture therapy combined with Chai Hu Shuo Liver San | Subjects were not tumour patients |  |
| 4 | Effect of Jiawei Jianzhong Tang combined with snap-needle treatment on Chinese medicine evidence score of patients with Hp-positive chronic gastritis | Subjects were not tumour patients |  |
| 5 | Clinical observation on the treatment of hiccups in advanced tumors by one-finger zen point pressure | The intervention group intervention not sole acupuncture |  |
| 6 | Observation on the effect of acupuncture point embedded needle combined with acupoint pressure in the treatment of tumors with spleen and stomach yang deficiency type of ergotism | duplicate document |  |
| 7 | Clinical observation of floating needle with reperfusion activity in the treatment of intractable ergotism | Subjects were not tumour patients |  |
| 8 | Carpal and ankle acupuncture combined with gastrofacial therapy in patients with recalcitrant eruption after radiotherapy for malignant tumors | duplicate document |  |
| 9 | Review of Traditional Chinese Medicine Treatment of Pancreatic Cancer | Review |  |
| 10 | The effects of acupuncture combined with Houpu exhaust syrup on gastrointestinal function, inflammatory factors and quality of life in patients undergoing radical surgery for cervical cancer | The intervention group intervention not sole acupuncture |  |
| 11 | Efficacy of esomeprazole and omeprazole in the treatment of Helicobacter pylori-associated gastric ulcer | Subjects were not tumour patients |  |
| 12 | Analysis of the effect of the combination of lansoprazole and traditional Chinese medicine in the treatment of spleen and stomach damp-heat type chronic erosive gastritis | Subjects were not tumour patients |  |
| 13 | Clinical study on the treatment of gastroparesis syndrome after gastrointestinal tumor surgery by acupuncture and foot-sanli combined with traditional Chinese medicine enema | The intervention group intervention not sole acupuncture |  |
| 14 | Liu Yaxian's “Stopping Vomiting Soup”: A clinical realization | review |  |
| 15 | A case of acupuncture in the treatment of nabulizumab-induced intractable eruption | case report |  |
| 16 | Clinical observation on the treatment of intractable ergotism after interventional therapy for primary hepatocellular carcinoma by combining yi qi and liver-reducing soup with acupuncture | The intervention group intervention not sole acupuncture |  |
| 17 | A case of pacemaker implantation for the treatment of atrial fibrillation with long RR intervals as the main symptom of ergospiration | case report |  |
| 18 | Analysis of the nursing effect of targeted care combined with metoclopramide injection at Neiguan acupoints on post-chemotherapy ergotherapy | The intervention group intervention not sole acupuncture |  |
| 19 | Clinical observation of esomeprazole combined with acupoint acupuncture in the treatment of gastroesophageal reflux disease of Zhongxu qi reversal type | The intervention group intervention not sole acupuncture |  |
| 20 | A study of clinical practice guidelines for the prevention and treatment of post-chemotherapy nausea with acupuncture | duplicate document |  |
| 21 | Clinical observation on the treatment of chronic non-atrophic gastritis with spleen and stomach deficiency and cold type by broken jade shunt needle array | Subjects were not tumour patients |  |
| 22 | Study of dexamethasone-induced ergospiration in patients with lymphoma treated with snap needles | duplicate document |  |
| 23 | Clinical observation on the treatment of intractable ergotism after radiotherapy for malignant tumors by penetrating point acupuncture method combined with traditional Chinese medicine | duplicate document |  |
| 24 | Observations on the efficacy of acupuncture in the treatment of advanced tumor eruption | Not RCT |  |
| 25 | Clinical study of acupuncture combined with acupoint injection for the treatment of post-chemotherapy ergotherapy in lung cancer | duplicate document |  |
| 26 | Umbilical and abdominal acupuncture combined with meridian discernment in the treatment of persistent ergotism in advanced hepatocellular carcinoma: perceptions | Personal Insights |  |
| 27 | Clinical study of abdominal acupuncture combined with scopolamine acupoint injection in the treatment of recalcitrant eruption after chemotherapy in 28 cases | duplicate document |  |
| 28 | Nursing effect of acupuncture intervention on intractable eruption after TACE in patients with hepatocellular carcinoma | duplicate document |  |
| 29 | Summary of Chief Physician Li Yi's Experience in Treating Ergotism Caused by Chemotherapy of Malignant Tumors | Personal Insights |  |
| 30 | Effects of Chaihu Shuohe San combined with Yanglingquan electroacupuncture on clinical symptoms and serum inflammatory cytokine levels in patients with chronic cholecystitis | The intervention group intervention not sole acupuncture |  |
| 31 | A review on the treatment of esophageal cancer by external therapeutic method of traditional Chinese medicine | Review |  |
| 32 | Therapeutic efficacy of warm acupuncture combined with Huang Lian Wen Bile Tang in the treatment of chronic superficial gastritis | The intervention group intervention not sole acupuncture |  |
| 33 | Clinical study on umbilical acupuncture combined with spleen-healthy and stomach-healthy soup in the treatment of chronic atrophic gastritis with weak spleen and stomach | The intervention group intervention not sole acupuncture |  |
| 34 | Analysis of the application effect of exhaust soup and auricular pressure bean therapy combined with body point snap acupuncture in postoperative abdominal distension after cesarean delivery | The intervention group intervention not sole acupuncture |  |
| 35 | Perioperative nursing experience of Angio-CT-guided hepatic artery embolization combined with microwave ablation for liver tumors | Personal Insights |  |
| 36 | Observation on the efficacy of traditional Chinese medicine combined with acupuncture in the treatment of tumor-associated intractable eruption | duplicate document |  |
| 37 | Clinical study on rapid-acting ergogenic flat soup combined with acupuncture in the treatment of intractable ergogenic deficiency after chemotherapy | duplicate document |  |
| 38 | Observation on the effect of acupuncture point embedded needle combined with acupoint pressure in the treatment of tumors with spleen and stomach yang deficiency type of ergotism | duplicate document |  |
| 39 | Progress in the treatment of intractable ergotism with traditional Chinese medicine | Review |  |
| 40 | Effect of warm acupuncture on stress response and immune function during radiotherapy in patients with malignant tumors of the digestive tract | First outcome indicator non-effective rate |  |
| 41 | Survey on sleep quality of postoperative patients with esophageal cancer and analysis of their influencing factors | Not RCT |  |
| 42 | Study on the efficacy and safety of treating chronic atrophic gastritis by nourishing yin and stomach and removing blood stasis soup combined with acupuncture therapy | The patient had no symptoms of hiccups. |  |
| 43 | Progress of acupuncture in the treatment of chemotherapy-induced eruption in malignant tumors | Review |  |
| 44 | Acupuncture with acupoint compresses in the treatment of intractable eruption in 30 cases | duplicate document |  |
| 45 | Acupuncture with acupoint injection of atropine in the treatment of recalcitrant eruption in tumor patients in 3 cases | duplicate document |  |
| 46 | Clinical study of acupressure combined with acupoint application in the treatment of recalcitrant eruption after chemotherapy | duplicate document |  |
| 47 | Exploring the care and application value of discomfort symptoms in patients with heat perfusion oncology treatment system under abdominal heat perfusion chemotherapy in the body cavity | The intervention group intervention not sole acupuncture |  |
| 48 | An overview of clinical studies on acupuncture in the treatment of intractable ergotism | Review |  |
| 49 | Clinical study of auricular acupuncture in the treatment of recalcitrant eruption after interventional therapy for ruptured hepatic tumor bleeding | Inclusion |  |
| 50 | Acupuncture treatment of 11 cases of intractable ergotism | duplicate document |  |
| 51 | Efficacy of baclofen in the treatment of recalcitrant eruption in patients undergoing radiotherapy for malignant tumors | duplicate document |  |
| 52 | Acupuncture in the treatment of intractable eruption due to tumor | duplicate document |  |
| 53 | A randomized parallel controlled study of clove persimmon tisane soup and acupoint injections combined with domperidone in the treatment of tumor-induced recalcitrant erysipelas | duplicate document |  |
| 54 | Treatment of 39 cases of leukopenia by acupuncture on large vertebrae and diaphragm yu | The patient had no symptoms of hiccups. |  |
| 55 | Clinical observation of traditional Chinese medicine identification and treatment combined with acupuncture and acupoint injection in the treatment of recalcitrant eruption after radiotherapy and chemotherapy | duplicate document |  |
| 56 | A case of spinning ochre soup combined with tangerine peel and bamboo rhizome soup with acupuncture and auricular acupoint pinching in the treatment of intractable hiccups in tumors. | duplicate document |  |
| 57 | Head acupuncture combined with body acupuncture in the treatment of 32 cases of intractable eruption after radiotherapy for malignant tumors | Not RCT |  |
| 58 | Retrospective investigation and analysis of the application of traditional Chinese medicine nursing operation techniques in the oncology department of a Chinese hospital | Not RCT |  |
| 59 | An investigation of the external treatment of chemotherapy-associated ergotism | Review |  |
| 60 | Clinical observation on acupuncture and moxibustion with acupoint injection in the treatment of intractable eruption of advanced malignant tumors | duplicate document |  |
| 61 | Clinical observation on the prevention and treatment of cisplatin chemotherapy-induced gastrointestinal symptoms by the old ten needles | duplicate document |  |
| 62 | The efficacy of phrenic nerve acupuncture stimulation in the treatment of recalcitrant eruption after intracranial tumor surgery | duplicate document |  |
| 63 | Observations on the efficacy of acupuncture in the treatment of tumor-associated ergotism | duplicate document |  |
| 64 | A case of acupuncture in the treatment of irritable bowel syndrome with anxiety | case report |  |
| 65 | Progress of Research Related to Acupuncture and Moxibustion for the Treatment of Tumors | Review |  |
| 66 | Clinical efficacy of acupoint injection in the treatment of recalcitrant ergotism in tumor patients | duplicate document |  |
| 67 | 26 cases of cancerous eruption treated by acupuncture of tianchu and massage of danzhong | duplicate document |  |
| 68 | A randomized parallel controlled study of Clove Persimmon Tee Soup + Hovenia Conducting Pill combined with metoclopramide foot-sanli acupoint injection for the treatment of intractable eruption of tumor chemotherapy | duplicate document |  |
| 69 | 1 case of esophageal sinus tract | case report |  |
| 70 | Clinical application of auricular acupoints in the diagnosis and treatment of tumors: a review | Review |  |
| 71 | Comparative study of acupuncture strong stimulation and acupoint injection in the treatment of cancer-related recalcitrant ergotism | duplicate document |  |
| 72 | Wrist and ankle acupuncture in the treatment of 25 cases of advanced gastric cancer with ergonomics | Not RCT |  |
| 73 | Internal administration of raw leek juice combined with acupuncture for the treatment of intractable eructation of upper gastrointestinal tract tumors | The intervention group intervention not sole acupuncture |  |
| 74 | Thirty-five cases of intractable ergotism treated with “old ten needles” and acupoint injections | Duplicate study |  |
| 75 | Adverse effects and care of patients after argon helium knife surgery for primary liver cancer | The intervention group intervention not sole acupuncture |  |
| 76 | Acupuncture therapy for postoperative gastroparesis syndrome: a case study | case report |  |
| 77 | Clinical observation and nursing care of ergotism in patients with chemotherapy for malignant tumors | Not RCT |  |
| 78 | Twenty-one cases of encephalopathy complicated by ergotism treated by acupuncture point submerged needling | not cancer patient |  |
| 79 | Clinical observation on the treatment of tumor-induced intractable ergotism by adding flavored clove persimmon tisane soup combined with acupoint injection | duplicate document |  |
| 80 | “Hammering therapy for the treatment of esophageal obstruction in cattle | Animal study |  |
| 81 | Acupuncture in the treatment of intractable eruption in cancer patients in 32 cases |  | Inclusion |
| 82 | Clinical experience of acupuncture in the treatment of 36 cases of cancerous intractable eruption | Not RCT |  |
| 83 | A study of acupuncture in the treatment of ergotism | Review |  |
| 84 | Care of patients in a phase I clinical trial of carmustine extended-release implants | Not RCT |  |
| 85 | The experience of warm acupuncture in the treatment of intractable ergotism | Not RCT |  |
| 86 | Care of patients with adverse reactions to nebulized inhalation after surgery for hepatocellular carcinoma | Not RCT |  |
| 87 | Hydro-acupuncture plus auricular acupressure in the treatment of recalcitrant eruption after tumor chemotherapy | The intervention group intervention not sole acupuncture |  |
| 88 | Electroacupuncture combined with auricular acupressure for the treatment of hiccups complicating advanced cancer(In English) | Duplicate study |  |
| 89 | Research progress of acupuncture to improve the side effects of antitumor therapy in cancer patients | Review |  |
| 90 | Acupuncture in the treatment of cancer-associated intractable eruption in 32 cases | Duplicate study |  |
| 91 | Acupuncture and moxibustion for the treatment of 25 cases of post-chemotherapy eruption of cancer | Not RCT |  |
| 92 | Therapeutic effect of acupuncture in treating 32 cases of ergotism after radiotherapy of tumor | Duplicate study |  |
| 93 | Observation on the efficacy of acupoint injection in the treatment of ergotism | The intervention group intervention not sole acupuncture |  |
| 94 | Electroacupuncture with acupressure in the treatment of intractable postoperative cancer eruption in 37 cases | The intervention group intervention not sole acupuncture |  |
| 95 | Overview of acupuncture treatment for ergotism in the last 10 years | Review |  |
| 96 | A randomized controlled trial of acupuncture for the treatment of gastroparesis after surgical abdominal oncology (English) | Patients have no symptoms of hiccups |  |
| 97 | Effects of cathartic and cognitive interventions on quality of life during chemotherapy in gastric cancer patients | Not RCT |  |
| 98 | Acupuncture and moxibustion in the treatment of cancer with intractable eruption in 28 cases | Not RCT |  |
| 99 | Nursing countermeasures for the development of ergotism after chemotherapy in cancer patients | Not RCT |  |
| Number | **CNKI Title** |  |  |
| 1 | Meta-analysis of the efficacy of acupoint stimulation therapy in the treatment of ergotherapy in patients with hepatocellular carcinoma | duplicate document |  |
| 2 | Clinical observation on the treatment of patients with recalcitrant eruption after intervention for primary hepatocellular carcinoma by acupuncture to the Zhiyang Bazhang acupoints combined with baclofen | duplicate document |  |
| 3 | The effect of snap-needle auricular points combined with acupoints in lung cancer chemotherapy-induced intractable ergotism | Inclusion |  |
| 4 | Therapeutic efficacy of submerged acupuncture at Zanzhu acupoint under reversed abdominal breathing in the treatment of chemotherapy-associated ergotism | The intervention group intervention not sole acupuncture |  |
| 5 | Improvement of postoperative ergotism in gastric cancer by acupressure points with foot reflexology | duplicate document |  |
| 6 | Clinical study of acupuncture combined with acupoint injection for the treatment of post-chemotherapy ergotherapy in lung cancer | duplicate document |  |
| 7 | Clinical study of abdominal acupuncture combined with scopolamine acupoint injection in the treatment of recalcitrant eruption after chemotherapy in 28 cases | duplicate document |  |
| 8 | Clinical study on the treatment of intractable hiccups after chemotherapy with the combination of Shu Xiao E Ni Ping Decoction and acupuncture for deficiency syndrome. | duplicate document |  |
| 9 | Clinical Observation on the Treatment of Refractory Hiccup Induced by Chemotherapy through the Integration of Traditional Chinese and Western Medicine | The intervention group intervention not sole acupuncture |  |
| 10 | Meta-analysis of the effect of traditional Chinese medicine acupoint application combined with acupoint massage on gastrointestinal reactions in cancer patients after chemotherapy | Meta analysis |  |
| 11 | Clinical study of acupuncture in the treatment of acute cerebral infarction resulting in central eruption | Subjects were not tumour patients |  |
| 12 | Analyzing the clinical efficacy of acupuncture in the treatment of intractable ergotism in Chinese medicine | conference |  |
| 13 | Therapeutic efficacy of Wu Zhu acupoint application combined with acupoint massage in the treatment of chemotherapy-associated ergotism | The intervention group intervention not sole acupuncture |  |
| 14 | Clinical observation on the treatment of intractable diaphragmatic spasm by acupuncture with drug acupoint injection | Subjects were not tumour patients |  |
| 15 | Clinical study on the treatment of intractable hiccups after chemotherapy by acupoint massage combined with acupoint application | duplicate document |  |
| 16 | Clinical Observation on the Therapeutic Effect of Ear Seed Pressure Combined with Acupuncture Therapy for Intractable Hiccups Induced by Malignant Tumor Radiochemotherapy | duplicate document |  |
| 17 | Observation on the Effect of Combined Salt Partition Moxibustion and Magnetic Needle Cupping at Zusanli Point in Preventing Gastrointestinal Reactions Caused by Chemotherapy in Nasopharyngeal Carcinoma Patients | duplicate document |  |
| 18 | Efficacy evaluation of raw persimmon soup and vitamin B acupoint injection in the treatment of intractable hiccups in tumor patients | The intervention group intervention not sole acupuncture |  |
| 19 | Observation on the efficacy of acupuncture combined with旋复代赭汤 in the treatment of intractable hiccups after chemotherapy with cisplatin regimen. | The intervention group intervention not sole acupuncture |  |
| 20 | Analyze the therapeutic effect of acupuncture treatment on tumor hiccups. |  | Inclusion |
| 21 | Observation on the Effect of Acupoint Injection of Metoclopramide Combined with Pressure on Yifeng Point for Intractable Hiccups Caused by Radiotherapy and Chemotherapy in Lung Cancer Patients | duplicate document |  |
| 22 | Clinical Observation on Acupuncture Combined with Acupoint Injection for the Treatment of Intractable Hiccup in Advanced Malignant Tumors | duplicate document |  |
| 23 | Clinical Observation on the Treatment of Intractable Hiccups after Chemoradiotherapy with TCM Syndrome Differentiation and Treatment Combined with Acupuncture and Point Injection | duplicate document |  |
| 24 | Randomized Parallel Control Study on the Treatment of Intractable Hiccup Caused by Tumors with Clove and Persimmon Stalk Decoction Combined with Acupoint Injection and Domperidone | duplicate document |  |
| 25 | Observation on the therapeutic effect of acupuncture in treating tumor hiccups | Inclusion |  |
| 26 | Clinical Observation on Relieving Hiccup in Chemotherapy Patients by Pressing the Shao Shang Acupoint | The intervention group intervention not sole acupuncture |  |
| 27 | Acupuncture treatment for diaphragm spasm | Subjects were not tumour patients |  |
| 28 | Clinical study on the treatment of intractable hiccups after gastrointestinal cancer surgery with acupoint electroacupuncture stimulation | duplicate document |  |
| 29 | Observation on the Efficacy of Acupuncture in Treating Tumor-Related Hiccups | duplicate document |  |
| 30 | Clinical efficacy of Bupleurum and Curcuma Decoction combined with acupuncture in the treatment of intractable hiccups after interventional therapy for liver cancer | duplicate document |  |
| 31 | Clinical Observation on the Therapeutic Effect of Acupoint Injection in Treating Refractory Hiccups in Tumor Patients | duplicate document |  |
| 32 | Observation on the Effect of Manual Massage Combined with Evodia rutaecarpa Paste Application at Yongquan Acupoint for the Treatment of Intractable Hiccups | duplicate document |  |
| 33 | Clinical Observation on the Treatment of Intractable Hiccups with the Combination of Chinese and Western Medicine and Acupuncture Therapy | The intervention group intervention not sole acupuncture |  |
| 34 | Clinical study on the treatment of intractable hiccups in cancer patients using traditional Chinese medicine combined with acupoint injection therapy. | duplicate document |  |
| 35 | Clinical Observation on the Treatment of Intractable Hiccup in Hematological Patients with Transcutaneous Electrical Acupuncture Point Stimulation | The intervention group intervention not sole acupuncture |  |
| 36 | Randomized Parallel Control Study on the Treatment of Refractory Hiccups from Tumor Chemotherapy with the Combination of Clove and Persimmon Stomach Decoction and Auricularis Triangularis Point Injection of Aurantii Fructus Immaturus and Chlorpromazine | duplicate document |  |
| 37 | Clinical Observation on the Treatment of Chemotherapy-induced Hiccups with Traditional Chinese Medicine Persimmon Calyx Decoction Combined with Acupoint Injection | The intervention group intervention not sole acupuncture |  |
| 38 | Clinical Observation on the Treatment of Intractable Hiccup Caused by Tumors with Clove and Persimmon Calyx Decoction Combined with Acupoint Injection | duplicate document |  |
| 39 | Observation on the Treatment of Intractable Hiccup in Patients with Severe Traumatic Brain Injury | Subjects were not tumour patients |  |
| 40 | Observation of the Efficacy of Comprehensive Nursing Intervention Combined with Doxepin Hydrochloride in the Treatment of Intractable Hiccups Following Radiotherapy and Chemotherapy | The intervention group intervention not sole acupuncture |  |
| 41 | Observation on the Effect of Acupressure Point Treatment on Hiccup after Interventional Therapy for Liver Cancer | duplicate document |  |
| 42 | Clinical Nursing of Acupuncture Treatment for Intractable Hiccup in 20 Cases of Upper Gastrointestinal Tumors | duplicate document |  |
| 43 | Acupressure at Zanzhu point combined with injection at Zusanli point for the treatment of intractable hiccups in 20 cases of malignant tumors. | duplicate document |  |
| 44 | Clinical Observation on the Efficacy of Electroacupuncture in Alleviating Gastrointestinal Adverse Reactions to Chemotherapy Drugs for Tumors | First outcome indicator non-effective rate |  |
| 45 | Observation on the Efficacy of Acupuncture for Intractable Hiccups after Interventional Chemotherapy for Liver Cancer |  | Inclusion |
| 46 | The application of acupressure at the Cuanzhu acupoint in the treatment of hiccups caused by chemotherapy | The intervention group intervention not sole acupuncture |  |
| 47 | Observation on the therapeutic effect of acupuncture combined with acupoint injection in the treatment of intractable hiccups after interventional therapy for liver cancer | duplicate document |  |
| 48 | The treatment of 56 cases of intractable hiccups in cancer patients with Wumo Yinzi combined with acupuncture. | duplicate document |  |
| 49 | Traditional Chinese Medicine Nursing for Hiccup Reactions in Patients Undergoing Chemotherapy | duplicate document |  |
| 50 | Clinical Observation of 47 Cases of Intractable Hiccup Treated with Electroacupuncture | Subjects were not tumour patients |  |
| 51 | Clinical Observation of 35 Cases of Intractable Hiccups Treated by Drug Injection at Zusanli Point | The intervention group intervention not sole acupuncture |  |
| 52 | Randomized Parallel Control Study on the Treatment of Refractory Hiccups from Tumor Chemotherapy with the Combination of Clove and Persimmon Stagnation Decoction and Auricularis Triangularis Point Injection of Trimebutine Maleate | duplicate document |  |
| 53 | Acupuncture combined with acupoint application therapy for 30 cases of intractable hiccups. | duplicate document |  |
|  |  |  |  |
| Number | **Wang Fang Title** | Exclude Reason |  |
| 1 | The application of wrist-ankle acupuncture combined with metoclopramide in the treatment of intractable hiccups in patients after malignant tumor radiochemotherapy. | Duplicate study |  |
| 2 | The application effect of wrist-ankle acupuncture in the treatment of intractable hiccups after interventional therapy for liver malignancy | Not RCT |  |
| 3 | Clinical Observation on the Treatment of Intractable Hiccup after Malignant Tumor Radiochemotherapy by Acupuncture at Acupoints Combined with Chinese Medicine | duplicate document |  |
| 4 | Observation on the Effect of Acupoint Embedding and Acupoint Pressing in the Treatment of Tumor-Induced Hiccup with Spleen-Stomach Yang Deficiency Type | duplicate document |  |
| 5 | Clinical Study on Persistent Hiccup after Gastrointestinal Cancer Surgery Treated with Electroacupuncture Stimulation at Acupoints | duplicate document |  |
| 6 | Study on Acupoint Application Combined with Acupuncture for Treating Hiccup After Transcatheter Arterial Embolization in Patients with Primary Liver Cancer | The intervention group intervention not sole acupuncture |  |
| 7 | Observation on the Effect of Acupoint Embedding Needle Combined with Acupoint Pressing in the Treatment of Tumor with Stomach-Spleen Yang Deficiency Type of Hiccup | duplicate document |  |
| 8 | Observation on the therapeutic effect of electroacupuncture and ear贴 on tumor-induced hiccups | duplicate document |  |
| 9 | Clinical Observation on Acupuncture Combined with Point Injection for the Treatment of Intractable Hiccup in Advanced Malignant Tumors | duplicate document |  |
| 10 | Observation on the Efficacy of Traditional Chinese Medicine Combined with Acupuncture in Treating Tumor-Related Intractable Hiccups | duplicate document |  |
| 11 | Observation on the therapeutic effect of electroacupuncture at Jiaji points for intractable hiccups after liver cancer resection surgery. |  | Inclusion |
| 12 | Observation on the therapeutic effect of acupuncture treatment for tumor hiccups | duplicate document |  |
| 13 | Clinical Observation on the Treatment of Intractable Hiccup in Patients with Primary Liver Cancer after Interventional Therapy by Acupuncture at the Eight Extraordinary Vessels Point of Shu Mansion Combined with Baclofen | duplicate document |  |
| 14 | Observation on the therapeutic effect of acupuncture in treating tumor hiccups | duplicate document |  |
| 15 | Acupuncture combined with acupoint injection therapy for intractable hiccups after interventional therapy for liver cancer | The intervention group intervention not sole acupuncture |  |
| 16 | Efficacy Observation of Baclofen in the Treatment of Intractable Hiccups in Patients with Malignant Tumors Undergoing Radiochemotherapy | duplicate document |  |
| 17 | Clinical Observation on Severe Hiccup after Interventional Postoperative Treatment of Liver Cancer with Acupuncture | The intervention group intervention not sole acupuncture |  |
| 18 | Clinical Observation on the Treatment of Refractory Hiccup after Transcatheter Arterial Embolization of Liver Cancer by Acupoint Application Combined with Acupuncture | The intervention group intervention not sole acupuncture |  |
| 19 | Analyze the therapeutic effect of acupuncture treatment on tumor hiccups. | duplicate document |  |
| 20 | Comparative study on acupuncture strong stimulation and acupoint injection therapy for cancer-related intractable hiccups | duplicate document |  |
| 21 | Meta-analysis of the therapeutic effect of acupoint stimulation therapy on hiccups in patients with liver cancer | duplicate document |  |
| 22 | Clinical Observation on the Treatment of Refractory Hiccup after Interventional Therapy for Liver Cancer by Acupuncture Combined with Traditional Chinese Medicine | The intervention group intervention not sole acupuncture |  |
| 23 | Clinical study on intractable hiccups after interventional treatment for ruptured liver tumor treated with auricular acupuncture | duplicate document |  |
| 24 | Clinical study on the treatment of intractable hiccups after chemotherapy with the combination of Shu Xiao E Ni Ping Decoction and acupuncture for deficiency syndrome. | duplicate document |  |
| 25 | The treatment of 56 cases of intractable hiccups in cancer patients with Wumo Yinzi combined with acupuncture. | duplicate document |  |
| 26 | Treatment of intractable hiccups in 28 cases with body acupuncture combined with ear acupoint pressure therapy. | The intervention group intervention not sole acupuncture |  |
| 27 | Clinical Observation on the Treatment of Intractable Hiccups after Chemoradiotherapy with TCM Syndrome Differentiation Combined with Acupuncture and Point Injection | duplicate document |  |
| 28 | Randomized Parallel Control Study on the Treatment of Intractable Hiccups Caused by Tumors with Clove and Persimmon Stalk Decoction Combined with Acupoint Injection and Domperidone | duplicate document |  |
| 29 | Randomized Parallel Control Study on the Treatment of Refractory Hiccups Induced by Tumor Chemotherapy with the Combination of Clove and Persimmon Stalk Decoction and Auricularis Triangularis Point Injection of Aurantii Fructus Immaturus and Chlorpromazine | duplicate document |  |
| 30 | Acupuncture combined with acupoint application in the treatment of 30 cases of intractable hiccups | duplicate document |  |
| 31 | Acupuncture treatment for intractable hiccups in 32 cancer patients | duplicate document |  |
| 32 | Observation on the Effect of Acupuncture Combined with Heat-Sensitive Moxibustion in the Treatment of Liver Cancer with Intractable Hiccups | The intervention group intervention not sole acupuncture |  |
| 33 | Clinical Study on the Treatment of Intractable Hiccups after Chemotherapy with Abdominal Acupuncture Combined with Anisodamine Acupoint Injection: A Report of 28 Cases | duplicate document |  |
| 34 | Observation on the therapeutic effect of acupuncture combined with modified XuanFu Dai Zhe Shi Tang for the treatment of intractable hiccups in advanced cancer patients. | duplicate document |  |
| 35 | Clinical Observation on Acupuncture Treatment for Hiccup in Liver Cancer Patients |  | Inclusion |
| 36 | Observation on the Nursing Effect of Acupuncture Intervention on Refractory Hiccup in Patients with Liver Cancer after TACE Surgery | duplicate document |  |
| 37 | Observational Study on the Treatment of Chemotherapy-Induced Hiccups with Flos Caryophylli and Pericarpium Citri Reticulatae Point Application | The intervention group intervention not sole acupuncture |  |
| 38 | Observation on the Efficacy of Acupuncture for Intractable Hiccups after Interventional Chemotherapy for Liver Cancer | duplicate document |  |
| 39 | Observation on the Effect of Acupoint Injection of Metoclopramide Combined with Pressure on Yifeng Point for Intractable Hiccups Caused by Radiotherapy and Chemotherapy in Lung Cancer Patients | duplicate document |  |
| 40 | Clinical Nursing of Acupuncture Treatment for Persistent Hiccup in 20 Cases of Upper Gastrointestinal Tumors | duplicate document |  |
| 41 | Randomized controlled trial of acupuncture for postoperative gastroparesis following abdominal tumor surgery | Patients have no symptoms of hiccups |  |
| 42 | Clinical Observation on the Therapeutic Effect of Acupoint Injection in Treating Intractable Hiccup in Tumor Patients | duplicate document |  |
| 43 | The effect of acupressure combined with foot reflexology on improving hiccups after gastric cancer surgery. | duplicate document |  |
| 44 | Acupressure at Zanzhu point combined with injection at Zusanli point for the treatment of intractable hiccups in 20 cases of malignant tumors. | duplicate document |  |
| 45 | Observation on the Efficacy of Combined Acupuncture and Traditional Chinese Medicine in Treating Refractory Hiccups in Tumor Patients | The intervention group intervention not sole acupuncture |  |
| 46 | Clinical Practice Guideline Research on Acupuncture for the Prevention and Treatment of Nausea After Chemotherapy | duplicate document |  |
| 47 | Clinical Observation on the Treatment of Intractable Hiccup after Gastric Cancer Surgery with Electroacupuncture |  | Inclusion |
| 48 | Observation on the Effect of Acupoint Massage Combined with Moxibustion for Relieving Qi Stagnation Type Hiccup in Patients After Liver Cancer Surgery | The intervention group intervention not sole acupuncture |  |
| 49 | Efficacy Observation of Ultrasound-guided Phrenic Nerve Block Combined with Water Injection in the Treatment of Intractable Hiccup after Chemoradiotherapy | not cancer patient |  |
| 50 | Observation on the Effect of Acupressure at Zanzhu Point for Treating Hiccups in Tumor Patients | The intervention group intervention not acupuncture |  |
| 51 | The application effect of acupoint pressing combined with acupoint therapy in the treatment of intractable hiccups caused by lung cancer chemotherapy. | duplicate document |  |
| 52 | Clinical efficacy of Bupleurum and Curcuma Decoction combined with acupuncture in the treatment of intractable hiccups after interventional therapy for liver cancer | duplicate document |  |
| 53 | Clinical Observation on the Treatment of Intractable Hiccup Caused by Tumors with Clove and Persimmon Calyx Decoction Combined with Acupoint Injection | duplicate document |  |
| 54 | Acupuncture treatment for intractable hiccups after chemotherapy and radiotherapy in 35 cases | duplicate document |  |
| 55 | The application of acupuncture therapy in patients with malignant tumor chemotherapy-resistant hiccups | Not RCT |  |
| 56 | Clinical Observation on the Prevention and Treatment of Gastrointestinal Reactions Caused by Chemotherapy through Acupoint Application of Evodia rutaecarpa Based on the Theory of Ziwu Liu Zhu | The intervention group intervention not sole acupuncture |  |
| 57 | Clinical Study of 35 Cases of Cancerous Intractable Hiccup Treated with Umbilical Acupuncture | Not RCT |  |
| 58 | Clinical Observation on the Treatment of Refractory Hiccup Caused by Tumor Radiotherapy with Huachansu Injection | duplicate document |  |
| 59 | Observation on the Efficacy of Acupuncture in Treating Tumor-Related Hiccups | duplicate document |  |
| 60 | Observation on the Effect of Acupoint Massage in Treating Hiccup in Patients with Gastric Cancer Complicated with Ascites and Pleural Effusion During Chemotherapy | The intervention group intervention not acupuncture |  |
| 61 | Integrated Traditional Chinese and Western Medicine Treatment and Nursing for Hiccup Induced by Chemotherapy in Cancer Patients | duplicate document |  |
| 62 | Observation on the therapeutic effect of acupoint injection at Zusanli for hiccup in 38 cases | The intervention group intervention not acupuncture |  |
| 63 | Meta-analysis of the effect of traditional Chinese medicine acupoint application combined with acupoint massage on gastrointestinal reactions in cancer patients after chemotherapy | duplicate document |  |
| 64 | Observation on the Effect of Acupressure Point Treatment on Hiccup after Interventional Therapy for Liver Cancer | duplicate document |  |
| 65 | Efficacy Evaluation of Traditional Chinese Medicine Shengshi Jiedu Decoction and Vitamin B1 Acupoint Injection on Refractory Hiccups in Tumor Patients | The intervention group intervention not acupuncture |  |
| 66 | Observation on the therapeutic effect of Zusanli acupoint injection of promethazine in the treatment of intractable vomiting after chemotherapy for hematological malignancies | The intervention group intervention not acupuncture |  |
| 67 | Observation on the Effect of Combined Salt Partition Moxibustion and Magnetic Needle Cupping at Zusanli Point in Preventing Gastrointestinal Reactions Induced by Chemotherapy in Nasopharyngeal Carcinoma Patients | duplicate document |  |
| 68 | Clinical study on the treatment of intractable hiccups in tumors with traditional Chinese medicine combined with acupoint injection | duplicate document |  |
| 69 | Observation on the Efficacy of Acupoint Injection Combined with Auricular Point Therapy for Intractable Hiccups in Patients with Advanced Liver Cancer | The intervention group intervention not acupuncture |  |
| 70 | Auricular point pressing needle and buried needle therapy for intractable hiccups | not cancer patient |  |
| 71 | The application of acupressure in alleviating chemotherapy-related nausea and vomiting in cancer patients | The intervention group intervention not acupuncture |  |
| 72 | Observation and Nursing of Acupoint Massage in Preventing Hiccup after Interventional Therapy for Liver Cancer | The intervention group intervention not acupuncture |  |
| 73 | Efficacy Analysis of Traditional Chinese Medicine Hot Compress Combined with Acupoint Massage in the Treatment of Hiccups After Esophageal Cancer Surgery | The intervention group intervention not acupuncture |  |
| 74 | Bilateral Zusanli point injection of atropine for the treatment of intractable hiccups after gastric surgery in 21 cases | The intervention group intervention not acupuncture |  |
| 75 | Clinical study on the treatment of intractable hiccups after chemotherapy by acupoint massage combined with acupoint application | duplicate document |  |
| 76 | Acupuncture Point Injection for the Treatment of Intractable Hiccups: A Report of 30 Cases | The intervention group intervention not acupuncture |  |
| 77 | Efficacy Observation of Acupoint Injection of Metoclopramide Combined with Dexmedetomidine Hydrochloride in the Treatment of Intractable Hiccups Induced by Chemotherapy | The intervention group intervention not acupuncture |  |
| 78 | Clinical Observation of 30 Cases of Intractable Hiccup Treated with Low-dose Chlorpromazine Acupoint Injection for Tumors | duplicate document |  |
| 79 | Study on the Application Effect of Indomethacin Suppositories Combined with Acupuncture in the Treatment of Postoperative Fever and Intractable Hiccup in Primary Liver Cancer Patients Undergoing TACE | The intervention group intervention not acupuncture |  |
| 80 | Clinical Analysis of 60 Cases of Intractable Hiccups Treated with Integrated Traditional Chinese and Western Medicine | The intervention group intervention not acupuncture |  |
| 81 | Acupuncture treatment for hiccups caused by dexamethasone in patients with lymphoma | duplicate document |  |
| 82 | Comparison of the Efficacy of Chlorpromazine and Ritalin in the Treatment of Intractable Hiccups and Nursing Care | The intervention group intervention not acupuncture |  |
| 83 | The application of evidence-based nursing in patients with liver cancer undergoing interventional chemotherapy. | The intervention group intervention not acupuncture |  |
| 84 | Construction and Application of an Adverse Reaction APP Continuation Care Model for Lung Cancer Chemotherapy Patients | Not RCT |  |
| 85 | Observation on the Efficacy of Holographic Massage Therapy in Treating Chemotherapy-Induced Nausea and Vomiting in Lung Cancer Patients | The intervention group intervention not acupuncture |  |
| 86 | Observation on the Effect of Manual Massage Combined with Evodia rutaecarpa Paste Application at Yongquan Acupoint for the Treatment of Intractable Hiccups | duplicate document |  |
| 87 | Clinical observation on the prevention and treatment of cisplatin chemotherapy-induced gastrointestinal symptoms by the old ten needles | duplicate document |  |
| 88 | The effect of dobutamine combined with traditional Chinese medicine treatment on inflammatory factors and matrix metalloproteinase function levels in children with severe pneumonia. | not cancer patient |  |
| 89 | Clinical Observation on the Treatment of Chronic Non-atrophic Gastritis with Modified Evodia and Areca Decoction and Acupoint Application | The intervention group intervention not acupuncture |  |
| 90 | Treatment of intractable hiccups in 42 cases with stomach-stabilizing injection at Zusanli acupoint. | The intervention group intervention not acupuncture |  |
| 91 | Study on the Recovery of Children with Bronchial Pneumonia Treated with Nano-Patch Application of Modified San'ao Decoction on Acupoints | The intervention group intervention not acupuncture |  |
| 92 | Clinical Observation on the Therapeutic Effect of Auricular Seed Pressing Combined with Acupuncture Therapy for Intractable Hiccups Induced by Malignant Tumor Radiochemotherapy | duplicate document |  |
| 93 | Clinical Observation on the Efficacy of Electroacupuncture in Alleviating Gastrointestinal Adverse Reactions to Chemotherapy Drugs for Tumors | duplicate document |  |
| 94 | Optimization of Treatment Strategies for Head and Neck Malignancies Oriented Towards Alleviating Damage Related to Chemoradiotherapy | Review |  |
| 95 | Clinical study on acupuncture at antiemetic acupoints for the treatment of chemotherapy-related nausea and vomiting | Patients have no symptoms of hiccups |  |
| 96 | Clinical study on nutritional status and quality of life assessment in patients with advanced gastric cancer | Review |  |
| 97 | Clinical Comparative Observation on the Auxiliary Treatment of Advanced Lung Cancer with Lung Infection by Using Tanreqing and Ambroxol Injections | The intervention group intervention not acupuncture |  |
| 98 | Screening for Prodromal Symptoms of Chemotherapy-Induced Vomiting in Lung Cancer Patients | Not RCT |  |
| 99 | The effect of N-acetylcysteine on oxidative stress, inflammation, and radiographic absorption in community-acquired pneumonia | not cancer patient |  |
| 100 | Meta-analysis of auricular acupressure for the treatment of nausea and vomiting caused by chemotherapy | Meta analysis |  |
| 101 | The effect and impact of隔药饼灸 (herbal cake moxibustion) intervention on chronic atrophic gastritis and the study of gene expression profiles in peripheral whole blood of CAG rats. | Animal study |  |
| 102 | Acupuncture combined with acupoint injection for the treatment of intractable hiccups after thoracoabdominal tumor surgery | The intervention group intervention not sole acupuncture |  |
| 103 | Observation on the therapeutic effect of electroacupuncture treatment for intractable hiccups in 35 patients with malignant tumors | duplicate document |  |
| 104 | Clinical Observation of Auricular Acupuncture Therapy in the Treatment of Malignant Tumor-Related Intractable Hiccups | Not RCT |  |
| 105 | Observation on the therapeutic effect of convenient acupuncture retention at body points for preventing postoperative hiccups in gastrointestinal tumors | The intervention group intervention not sole acupuncture |  |
| 106 | Clinical Observation on Acupuncture Treatment for Intractable Hiccups Caused by Malignant Tumors | Not RCT |  |
| 107 | Clinical Research Progress on Acupuncture for the Improvement of Nausea and Vomiting after Chemotherapy | Review |  |
| 108 | Observation of Clinical Efficacy of Ziwu Liu Zhu Low Frequency Therapeutic Instrument in the Treatment of Advanced Liver Cancer | The intervention group intervention not acupuncture |  |
| 109 | Electroacupuncture treatment for hiccups in 49 cases after tumor radiotherapy and chemotherapy. | duplicate document |  |
| 25 | Acupuncture Point Injection for Malignancy-Associated Hiccup: A Case Report | case report |  |
| Number | **VIP Title** | Exclude Reason |  |
| 1 | Clinical Observation on Acupuncture Combined with Point Injection for the Treatment of Intractable Hiccup in Advanced Malignant Tumors | duplicate document |  |
| 2 | Clinical Observation on the Treatment of Intractable Hiccup in Tumor Patients with Acupuncture at the Eight Convergent Points of the Cheng's Meridians | The intervention group intervention not sole acupuncture |  |
| 3 | Clinical Observation on the Treatment of Intractable Hiccup after Malignant Tumor Radiochemotherapy by Acupuncture at Acupoints Combined with Chinese Medicine | duplicate document |  |
| 4 | Randomized Parallel Control Study on the Treatment of Intractable Hiccups from Tumor Chemotherapy with the Combination of Clove and Persimmon Stagnation Decoction and Auricularis Triangularis Point Injection of Aurantii Fructus Immaturus and Metoclopramide | duplicate document |  |
| 5 | Randomized Parallel Control Study on the Treatment of Intractable Hiccups Caused by Tumors with Clove and Persimmon Stalk Decoction Combined with Acupoint Injection and Domperidone | duplicate document |  |
| 6 | Acupuncture combined with acupoint injection of atropine for the treatment of intractable hiccups in three cases of cancer patients. | duplicate document |  |
| 7 | Observation on the Effect of Acupoint Embedding and Acupoint Pressing in the Treatment of Tumor-Induced Hiccup with Spleen-Stomach Yang Deficiency Type | duplicate document |  |
| 8 | A case report on the treatment of intractable hiccups in cancer patients using the combination of Xuanfudai Zhe Decoction with Jupi Zhuru Decoction, along with acupuncture and ear point pressing. | duplicate document |  |
| 9 | Observation on the therapeutic effect of electroacupuncture and ear贴 on tumor-induced hiccups | duplicate document |  |
| 10 | Observation on the Efficacy of Acupuncture in Treating Tumor-Related Hiccups | duplicate document |  |
| 11 | The application of acupuncture in the treatment of intractable hiccups caused by tumors. | duplicate document |  |
| 12 | Observation on the Effect of Acupoint Embedding and Acupoint Pressing in the Treatment of Tumor-Induced Hiccup with Spleen and Stomach Yang Deficiency Type | duplicate document |  |
| 13 | Electroacupuncture treatment for hiccups in 49 cases after tumor radiotherapy and chemotherapy | duplicate document |  |
| 14 | Observation on the therapeutic effect of phrenic nerve acupuncture stimulation in the treatment of intractable hiccups after intracranial tumor surgery | duplicate document |  |
| 15 | Clinical Nursing of Acupuncture Treatment for Intractable Hiccup in 20 Cases of Upper Gastrointestinal Tumors | duplicate document |  |
| 16 | Clinical Observation on the Treatment of Refractory Hiccup after Chemoradiotherapy by Integrating Traditional Chinese Medicine Syndrome Differentiation with Acupuncture and Point Injection | duplicate document |  |
| 17 | Clinical Observation of 30 Cases of Intractable Hiccup Treated with Low-dose Chlorpromazine Acupoint Injection for Tumors | duplicate document |  |
| 18 | Clinical Observation on the Therapeutic Effect of Acupoint Injection in Treating Refractory Hiccup in Tumor Patients | duplicate document |  |
| 19 | Clinical efficacy of acupoint massage combined with Evodia rutaecarpa application at Yongquan acupoint in treating hiccups in tumor patients. | duplicate document |  |
| 20 | Clinical Observation on the Treatment of Intractable Hiccup Caused by Tumors with Clove and Persimmon Calyx Decoction Combined with Acupoint Injection | duplicate document |  |
| 21 | Observation on the Effect of Oral Modified Clove and Persimmon Calyx Decoction Combined with Acupoint Injection of Anisodamine in the Treatment of Malignancy-Related Hiccups | The intervention group intervention not sole acupuncture |  |
| 22 | The treatment of 56 cases of intractable hiccups in cancer patients with Wumo Yinzi combined with acupuncture. | duplicate document |  |
| 23 | Acupressure at Zanzhu point combined with injection at Zusanli point for the treatment of intractable hiccups in 20 cases of malignant tumors. | duplicate document |  |
| 24 | Atropine acupoint injection combined with moxibustion therapy for intractable hiccups in 33 cases of advanced malignant tumors. | duplicate document |  |
| 25 | Acupuncture Point Injection for Malignancy-Associated Hiccup: A Case Report | case report |  |
| 26 | The effect of acupoint injection intervention on hiccups and quality of life in patients with malignant brain metastases | The intervention group intervention not sole acupuncture |  |
| 27 | Observation on the therapeutic effect of acupoint injection with Metoclopramide Injection for the treatment of intractable hiccups in advanced cancer patients | The intervention group intervention not sole acupuncture |  |
| 28 | Acupuncture point application combined with ear point pressure for the treatment of intractable hiccups in 65 cancer patients. | The intervention group intervention not sole acupuncture |  |
| 29 | Efficacy evaluation of raw persimmon soup and vitamin B1 acupoint injection in the treatment of intractable hiccups in cancer patients | The intervention group intervention not sole acupuncture |  |
| 30 | Clinical study on the treatment of intractable hiccups in tumors with traditional Chinese medicine combined with acupoint injection | duplicate document |  |
| 31 | Experiences in Acupuncture Treatment for 11 Cases of Intractable Hiccups | duplicate document |  |
| 32 | Acupuncture Point Injection for Refractory Hiccup in 26 Cases of Advanced Cancer | The intervention group intervention not sole acupuncture |  |
| 33 | Acupuncture Point Injection for the Treatment of Intractable Hiccups in 22 Cases | The intervention group intervention not sole acupuncture |  |
| 34 | Observation on the Efficacy of Acupuncture for Intractable Hiccups after Interventional Chemotherapy for Liver Cancer | duplicate document |  |
| 35 | Observation on the therapeutic effect of electroacupuncture at Jiaji points for intractable hiccups after liver cancer resection surgery | duplicate document |  |
| 36 | Acupuncture treatment for intractable hiccups | Review |  |
| 37 | Acupuncture at Tiantu combined with acupressure at Danzhong for the treatment of 26 cases of cancerous hiccups. | duplicate document |  |
| 38 | Observation and Treatment of Hiccup in Neurosurgical Patients | not cancer patient |  |
| 39 | Construction and Application of an Adverse Reaction APP Continuation Care Model for Lung Cancer Chemotherapy Patients | duplicate document |  |
| Number | **Pubmed Title** | Exclude Reason |  |
| 1 | Acupuncture for cancer patients suffering from hiccups: a systematic review and meta-analysis | Review |  |
| 2 | Acupuncture for cancer-related hiccups: Protocol for a systematic review and meta-analysis of randomized controlled trials | Review |  |
| 3 | Effectiveness of acupuncture and related therapies for palliative care of cancer: overview of systematic reviews | Review |  |
| 4 | [Therapeutic effect of cervical Jiaji electroacupuncture on postoperative intractable hiccup of liver neoplasms] | duplicate document |  |
| 5 | [Clinical observation on yintang (Ex-HN 3) point-injection for treatment of obstinate hiccup] | The intervention is not acupuncture |  |
| 6 | Acupuncture treatment for 57 cases of hiccup | Not RCT |  |
| 7 | Pharmacopuncture for cancer care: a systematic review | Review |  |
| 8 | Hiccups in patients with cancer: a multi-site, single-institution study of etiology, severity, complications, interventions, and outcomes | Not RCT |  |
| 9 | [Spectrum and indications of acupuncture and moxibustion therapy based on bibliometric analysis] | Biliometric analysis |  |
| 10 | Twenty-five cases of obstinate hiccup treated by point injection at tianding | case report |  |
| 11 | Chronic Hiccups: An Underestimated Problem | review |  |
| 12 | [Analysis on characteristics and regularities of efficacies of acupoint injection by using data mining technique] | Review |  |
| 13 | Study of wrist-ankle acupuncture therapy for optimizing anaesthesia scheme of painless gastroscopy and improving painless gastroscopy related complications | Subjects are not cancer patients |  |
| Number | **Web of science Title** | Exclude Reason |  |
| 1 | Hiccup and neurosurgeons: a report of 4 rare dorsal medullary compressive pathologies and review of the literature | report |  |
| 2 | Acupuncture treatment for 57 cases of hiccup | duplicate document |  |
| 3 | Acupuncture for cancer patients suffering from hiccups: A systematic review and meta-analysis | duplicate document |  |
| 4 | [Acupuncture on Tiantu (CV 22) combined with pressing acupiont Danzhong (CV 17) for 26 cancer patients suffering from hiccup] | duplicate document |  |
| 5 | Hiccups in patients with cancer: a multi-site, single-institution study of etiology, severity, complications, interventions, and outcomes | Review |  |
| 6 | Acupuncture Treatment for Persistent Hiccups in Patients with Cancer | Review |  |
| 7 | Acupuncture for cancer-related hiccups Protocol for a systematic review and meta-analysis of randomized controlled trials | protocol |  |
| 8 | Peppermint Inhalation Therapy for Persistent Hiccups in Terminally Ill Cancer Patient: A Case Study | case study |  |
| 9 | Effectiveness of acupuncture and related therapies for palliative care of cancer: overview of systematic reviews | overview |  |
| Number | **Embase Title** | Exclude Reason |  |
| 1 | Amantadine for the intractable hiccup when weaning a patient with a brainstem lesion-a case study | case report |  |
| 2 | Phrenic nerve block combined with stellate ganglion block for postoperative intractable hiccups: a case report | case report |  |
| 3 | Electroacupuncture vs Sham Electroacupuncture in the Treatment of Postoperative Ileus after Laparoscopic Surgery for Colorectal Cancer: A Multicenter, Randomized Clinical Trial | Not RCT |  |
| 4 | Hiccups in patients with cancer: a multi-site, single-institution study of etiology, severity, complications, interventions, and outcomes | Not RCT |  |
| 5 | Spectrum and indications of acupuncture and moxibustion therapy based on bibliometric analysis | Bibliometric analysis |  |
| 6 | Erratum regarding missing Declaration of Competing Interest statements in previously published articles (World Journal of Acupuncture – Moxibustion (2019) 29(3) (206–209), (S1003525719300583), (10.1016/j.wjam.2019.05.009)) | Review |  |
| 7 | Case 9-2021: A 16-year-old boy with headache, abdominal pain, and hypertension | case report |  |
| 8 | Development trend and current situation of acupuncture-moxibustion indications | Review |  |
| 9 | Acupuncture for cancer-related hiccups: Protocol for a systematic review and meta-Analysis of randomized controlled trials | Protocol |  |
| 10 | Hiccup after chemotherapy for lung cancer | Review |  |
| 11 | Acupoint selection pattern of chronic atrophic gastritis based on data mining methods of latent structure model and frequency item set | Review |  |
| 12 | Therapeutic effect of cervical Jiaji electroacupuncture on postoperative intractable hiccup of liver neoplasms | duplicate document |  |
| 13 | Effects of Acupuncture, Tuina, Tai Chi, Qigong, and Traditional Chinese Medicine Five-Element Music Therapy on Symptom Management and Quality of Life for Cancer Patients: A Meta-Analysis | Meta analysis |  |
| 14 | Effectiveness of acupuncture and related therapies for palliative care of cancer: Overview of systematic reviews | duplicate document |  |
| 15 | Intranasal vinegar as the effective treatment for persistent hiccups in a patient with advanced cancer patientundergoing palliative care: A case report | case report |  |
| 16 | A case report of postsurgical gastroparesis | case report |  |
| 17 | Effectiveness of acupuncture and related therapies for palliative care of cancer: overview of systematic reviews | duplicate document |  |
| 18 | Pharmacopuncture for cancer care: A systematic review | duplicate document |  |
| 19 | [Acupuncture on Tiantu (CV 22) combined with pressing acupiont Danzhong (CV 17) for 26 cancer patients suffering from hiccup]. | duplicate document |  |
| 20 | Hiccup: Mystery, nature and treatment | Review |  |
| 21 | Traditional chinese medicine in cancer care: A review of case series published in the chinese literature | Review |  |
| 22 | Acupuncture for chemotherapy-induced nausea and vomiting: A retrospective review of patients' experience at a single institution and a review of the literature | Review |  |
| 23 | Evolving treatment paradigms for chemotherapy-induced nausea and vomiting | The intervention group intervention not acupuncture |  |
| 24 | Palliative care of people with oesophageal cancer | Not RCT |  |
| 25 | Palliating hiccups in cancer patients: Moving beyond recommendations from Leonard the Lion | Review |  |
| 26 | Managing nausea and vomiting in palliative care: A practical guide | guide |  |
| 27 | Towards a Model for Planning Clinical Research in Oriental Medicine | Review |  |
| 28 | The Most Addictive Drug, the Most Deadly Substance: Smoking Cessation Tactics for the Busy Clinician | Not RCT |  |
| 29 | The management of hiccups in advanced cancer | Not RCT |  |
| 30 | Lymphoma - A personal experience | personal experience |  |
| Number | **The Cochrane Library Title** | Exlclude Reason |  |
| 1 | Therapeutic effect of cervical Jiaji electroacupuncture on postoperative intractable hiccup of liver neoplasms | duplicate document |  |
| 2 | The effect of auricular acupressure seed therapy on the recovery time of gastrointestinal function and clinical symptom scores in patients undergoing laparoscopic radical gastrectomy. | The intervention is not acupuncture |  |
