# Supplementary material for: Effectiveness and safety of acupuncture for cancer-related hiccups: a systematic review and meta-analysis
Source: Front Neurol. 2024 Dec 11;15:1480656. doi: 10.3389/fneur.2024.1480656 (PMC11668674; doi:10.3389/fneur.2024.1480656)
Supplement: Supplementary file 2 [file Supplementary_file_2.docx]

**Sensitivity analysis of overall effectiveness rate**

**
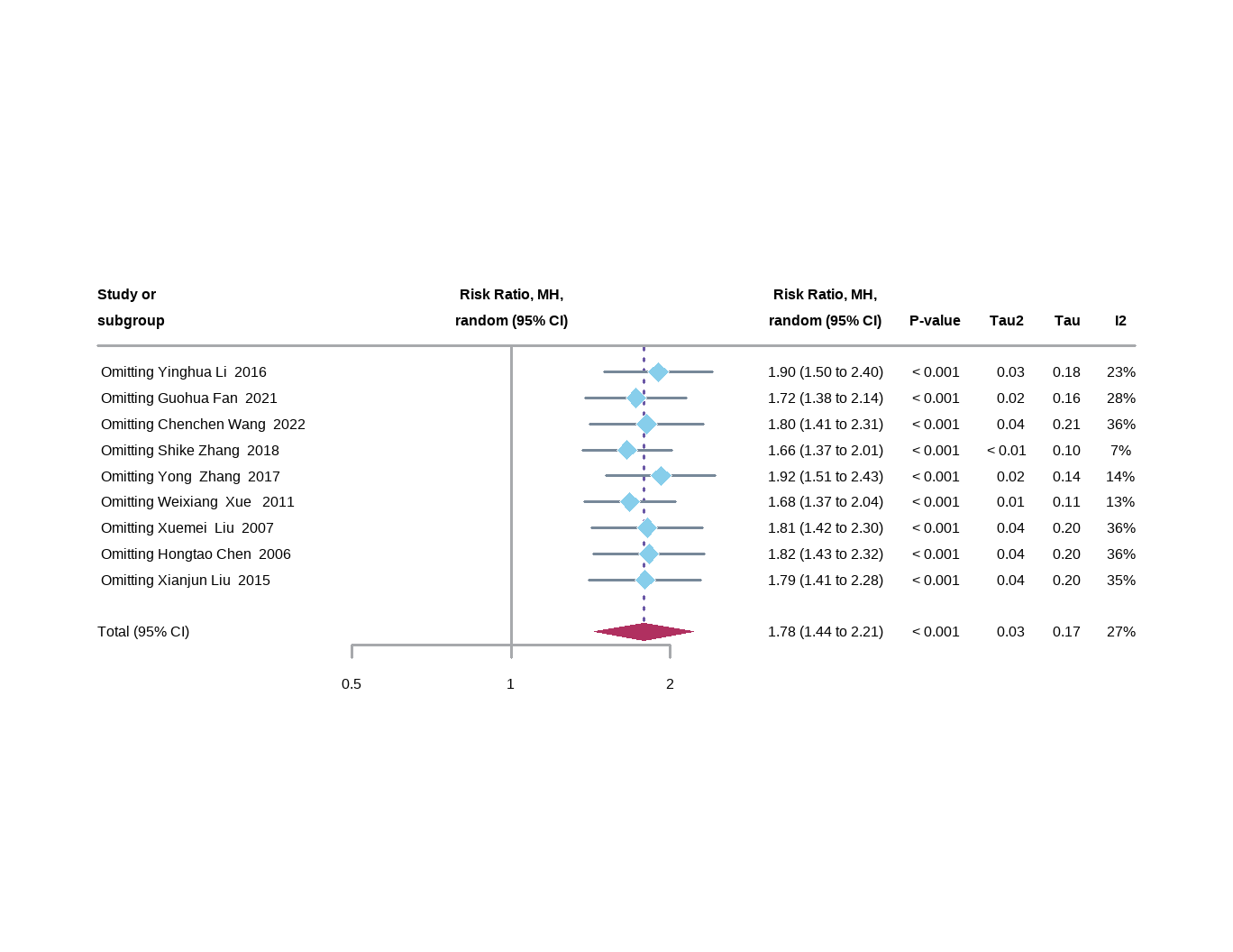
**

**Sensitivity analysis of quality of life**


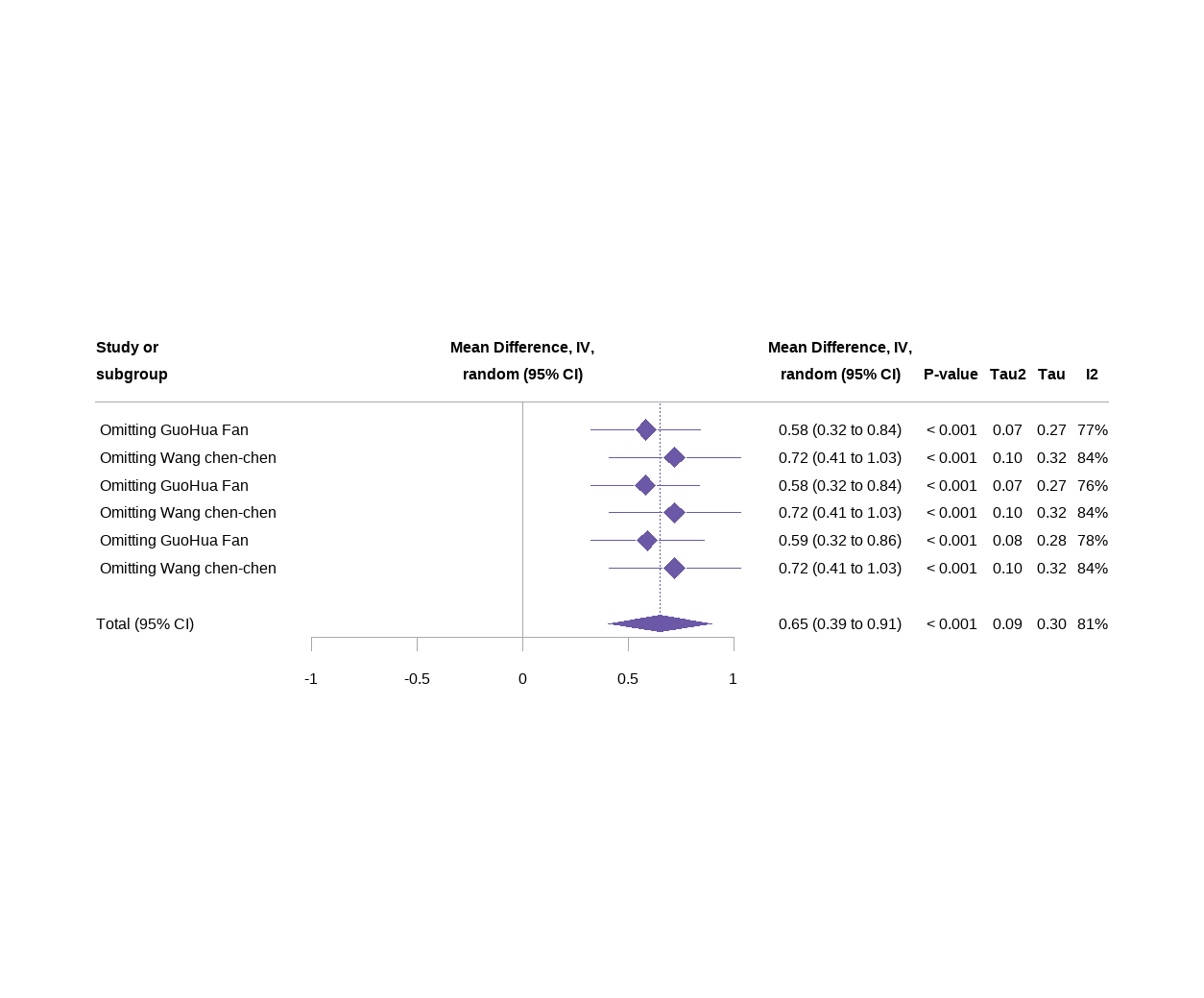


**Sensitivity analysis of onset time**


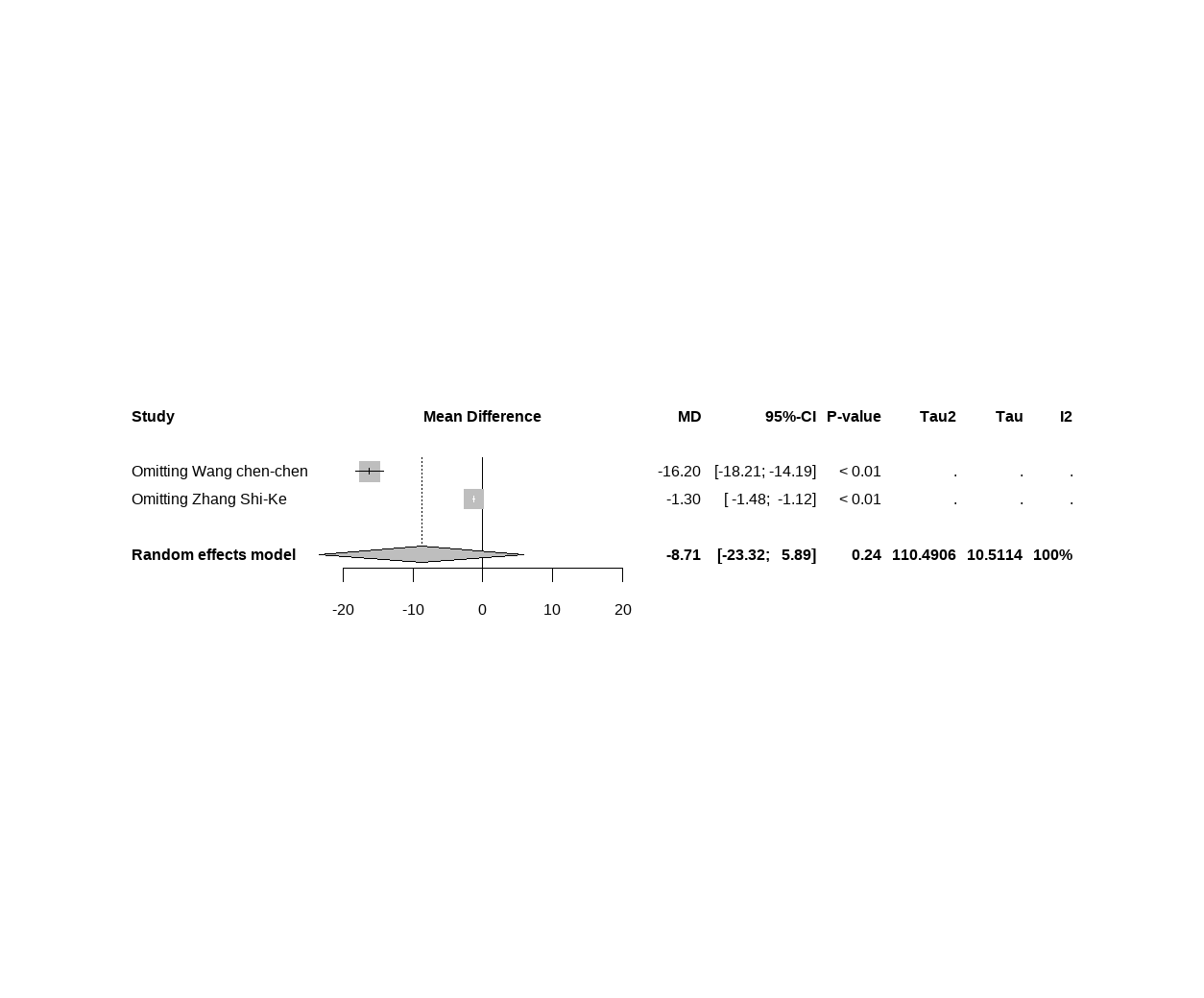


**Meta regression of effective rate**

metareg(metadata,Type)

Mixed-Effects Model (k = 6; tau^2 estimator: REML)

tau^2 (estimated amount of residual heterogeneity): 0 (SE = 0.0010)

tau (square root of estimated tau^2 value): 0

I^2 (residual heterogeneity / unaccounted variability): 0.00%

H^2 (unaccounted variability / sampling variability): 1.00

R^2 (amount of heterogeneity accounted for): 100.00%

Test for Residual Heterogeneity:

QE(df = 4) = 0.0137, p-val = 1.0000

Test of Moderators (coefficient 2):

QM(df = 1) = 25.7135, p-val < .0001

Model Results:

estimate se zval pval ci.lb ci.ub

intrcpt 0.4000 0.0182 21.9381 <.0001 0.3643 0.4357 ***

Type QA 0.6200 0.1223 5.0708 <.0001 0.3804 0.8597 ***

Signif. codes: 0 ‘***’ 0.001 ‘**’ 0.01 ‘*’ 0.05 ‘.’ 0.1 ‘ ’ 1
